# Biochar Decelerates Soil Organic Nitrogen Cycling but Stimulates Soil Nitrification in a Temperate Arable Field Trial

**DOI:** 10.1371/journal.pone.0086388

**Published:** 2014-01-30

**Authors:** Judith Prommer, Wolfgang Wanek, Florian Hofhansl, Daniela Trojan, Pierre Offre, Tim Urich, Christa Schleper, Stefan Sassmann, Barbara Kitzler, Gerhard Soja, Rebecca Clare Hood-Nowotny

**Affiliations:** 1 Department of Microbiology and Ecosystem Science, University of Vienna, Vienna, Austria; 2 Department of Ecogenomics and Systems Biology, University of Vienna, Vienna, Austria; 3 Core Facility of Cell Imaging and Ultrastructure Research, University of Vienna, Vienna, Austria; 4 Institute of Forest Ecology and Soil, Federal Research and Training Centre for Forests, Natural Hazards and Landscape, Vienna, Austria; 5 Department of Health and Environment, Austrian Institute of Technology, Tulln, Austria; University of Missouri, United States of America

## Abstract

Biochar production and subsequent soil incorporation could provide carbon farming solutions to global climate change and escalating food demand. There is evidence that biochar amendment causes fundamental changes in soil nutrient cycles, often resulting in marked increases in crop production, particularly in acidic and in infertile soils with low soil organic matter contents, although comparable outcomes in temperate soils are variable. We offer insight into the mechanisms underlying these findings by focusing attention on the soil nitrogen (N) cycle, specifically on hitherto unmeasured processes of organic N cycling in arable soils. We here investigated the impacts of biochar addition on soil organic and inorganic N pools and on gross transformation rates of both pools in a biochar field trial on arable land (Chernozem) in Traismauer, Lower Austria. We found that biochar increased total soil organic carbon but decreased the extractable organic C pool and soil nitrate. While gross rates of organic N transformation processes were reduced by 50–80%, gross N mineralization of organic N was not affected. In contrast, biochar promoted soil ammonia-oxidizer populations (bacterial and archaeal nitrifiers) and accelerated gross nitrification rates more than two-fold. Our findings indicate a de-coupling of the soil organic and inorganic N cycles, with a build-up of organic N, and deceleration of inorganic N release from this pool. The results therefore suggest that addition of inorganic fertilizer-N in combination with biochar could compensate for the reduction in organic N mineralization, with plants and microbes drawing on fertilizer-N for growth, in turn fuelling the belowground build-up of organic N. We conclude that combined addition of biochar with fertilizer-N may increase soil organic N in turn enhancing soil carbon sequestration and thereby could play a fundamental role in future soil management strategies.

## Introduction

Carbon farming opportunities in temperate agricultural systems are limited to no-till farming and conservation agriculture strategies. In these systems careful carbon management is required to avoid problems of nutrient, particularly N, immobilization by the soil microbial community, which can lead to substantial crop yield depressions. Biochar application to temperate agricultural soils offers the prospect for substantial carbon sequestration without the concomitant yield losses or greenhouse gas emissions associated with other strategies [1]–[4]. The pyrolysis derived aromatic-macromolecular structure of biochar [5] makes it more recalcitrant to microbial decomposition, which means it does not immobilize significant quantities of valuable nutrients when added to soil, rendering biochar a viable long-term carbon sink option for temperate agriculture [6]–[7]. Promoting novel carbon farming options in a wider agronomic context requires facing risk-benefit assessments by the soil custodians, namely farmers and other environmentally concerned stakeholders. As a result, an in-depth understanding of the impact of biochar on the soil N cycle, the most economically relevant biogeochemical cycle to farmers in mid to high latitudes, will be required to facilitate decision-making and allow for improved biochar management and subsequent adoption as a carbon sequestering strategy.

There is increasing evidence suggesting that biochar addition to soil impacts a number of processes of the soil N cycle [8] and that the impacts of biochar addition are strongly dependent on the biochar feedstock and the pyrolysis conditions, in addition to the respective soil properties and local environmental and climatic conditions [9]. For instance, biochar was shown to decrease nitrate leaching [10]–[11], to increase soil N immobilization [12]–[13], to increase ammonification, the conversion of organic N into ammonium [14]–[15], to increase nitrification, the oxidation of ammonium to nitrate [16]–, to promote volatilization, the loss of ammonia to the atmosphere [18], and to increase plant N uptake [19]–[21]. The mechanisms posited to explain the differences in N cycling processes when biochar is added to soil are mostly direct ones, resulting from the unique properties of the biochar such as its highly porous structure, the large surface area and its ion-exchange capacity [22], with associated beneficial effects on nutrient retention and water holding capacity. Some N cycle processes like NH_3_ volatilization can be stimulated by inherent biochar properties such as the potential of alkaline biochar to increase soil pH [23]. Moreover biochars can have high nutrient contents and increase soil nutrient availability directly or through the effects of priming which may increase the bio-availability of soil nutrients [24]–[25]. In addition, indirect mechanisms associated with changes in the soil microbial community can be triggered by the properties specific to biochar and thereby have strong implications for soil microbial N processing [16], [26]. To date the majority of biochar studies were carried out as short-term pot experiments, many of these focusing on poor, acidic and nutrient depleted soils where the addition of biochar has the potential to cause largest effects including fundamental changes in soil nutrient cycles, often resulting in pronounced increases in crop production [27]–[28]. Long-term field trials examining the effects of biochar on intensively managed and fertile agricultural soils in temperate regions are therefore urgently needed to advance our predictive capabilities of biochar - soil N cycle interactions [8], [20].

**Figure 1:**
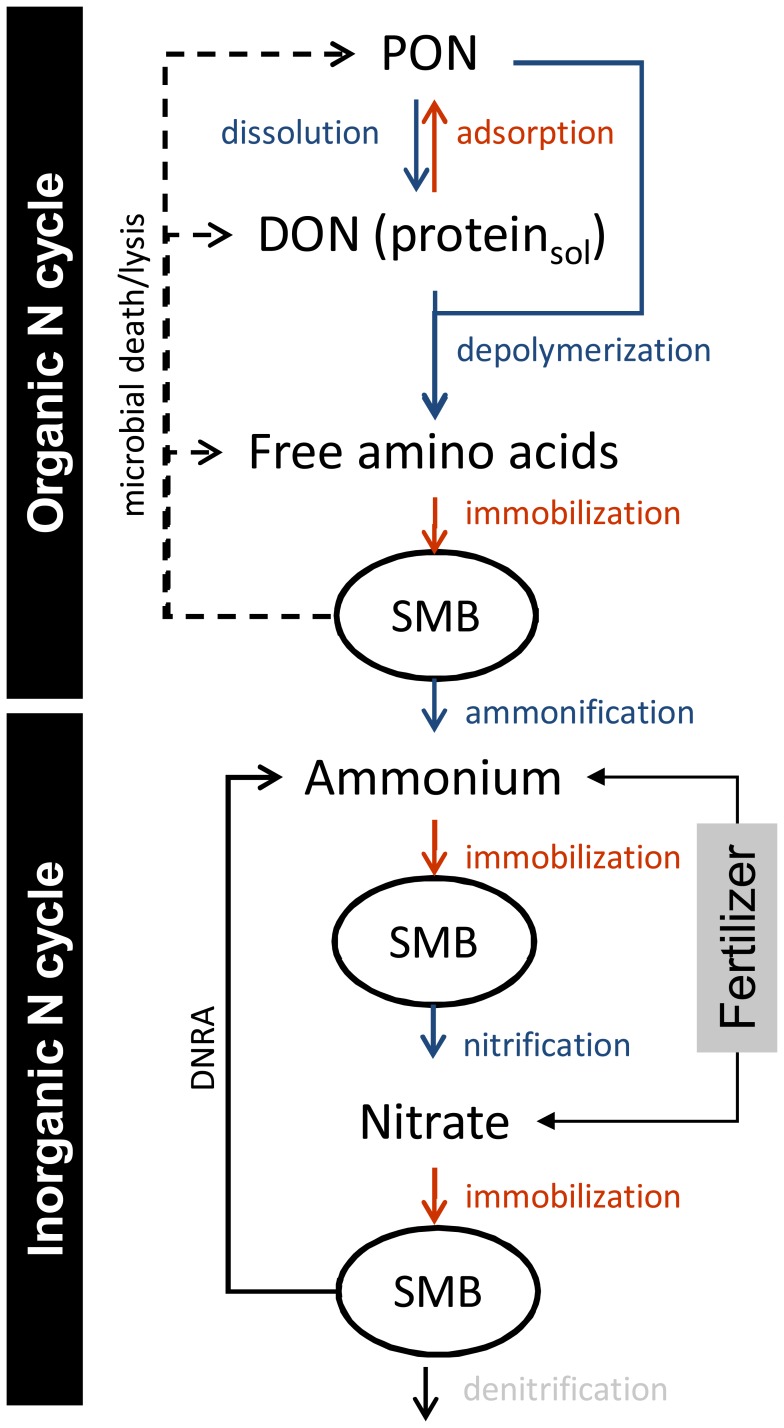
Overview of the agricultural soil N cycle. The organic N and inorganic N cycles are highlighted. DNRA…dissimilatory nitrate reduction to ammonium, DON…dissolved organic N, PON…particulate organic N such as detritus and soil organic matter, SMB…soil microbial biomass. Not shown - heterotrophic nitrification which results from microbial uptake of e.g. free amino acids, oxidation of reduced N compounds and release of nitrate into the soil.

The impacts of biochar on soil N transformation processes are extremely important, given the dominant role N nutrition plays in regulating crop production in temperate regions, as it has to be replenished either as inorganic N fertilizer or it is cycled back to the soil in a complex organic form such as crop residues. In the past two decades there has been a major paradigm shift in our conceptualization of the soil N cycle [29]. It places greater emphasis on the organic turnover processes in the soil N cycle such as the depolymerization of organic matter, which is now regarded as the rate limiting step of dissolved organic N production and subsequently of soil N mineralization and nitrification. As a result of the biochar characteristics described above biochar addition to soil could have major impacts on the organic N turnover component of the soil N cycle. The multitude of simultaneously occurring soil N processes (Figure 1) make measuring and understanding soil N transformation rates difficult and thus requires isotopic tools [30]–[31]. Using an array of parallel established and novel ^15^N isotope pool dilution techniques [32]–[33], it should be possible to study each key transformation process independently. These techniques in consortium allow determining the impact of biochar on multiple soil processes in a synchronized manner and with a high degree of resolution and reliability. In the context of biochar addition this issue of complexity is of particular relevance as biochar addition could have conflicting impacts on different control points of the soil N cycle.

Our major objective therefore was to investigate the effects of biochar on key inorganic and organic N transformation processes, on soil C and N pools and on the soil microbial community. A hardwood-based biochar (pyrolysis at 500°C for two hours) was added in March 2011 to an arable field on calcareous Chernozem in Traismauer, Austria. The field trial consisted of four treatments replicated four times in circular plots of 6.5 m, comprising NPK fertilized plots as controls combined with biochar addition rates of 0, 24 and 72 t dry mass ha^−1^. As agricultural systems rely on fertilization to grant crop production, fertilized plots were taken as controls (NPK, no biochar) and compared to the two biochar addition rates i.e. 24 t BC ha^−1^ with NPK amendment (BC1N) and 72 t BC ha^−1^ with NPK amendment (BC3N). A treatment with 72 t BC ha^−1^ lacking NPK amendment was also setup (BC3) to check for nutrient immobilization effects of BC-alone treatments. In this study we focused on the control (NPK) and the highest biochar treatment (BC3N). Soil samples were taken four months after biochar application and sampling was repeated several times during a period of fourteen months. We applied a novel array of isotope pool dilution assays to measure gross rates of protein, amino acid, ammonium and nitrate dynamics (Figure 1) and molecular techniques to investigate nitrifier (ammonium oxidizer) population sizes. In addition we used scanning electron microscopy to examine the physical structure of the biochar in order to understand its possible role in shaping the soil microbial community.

## Results

### Biochar Porosity

**Figure 2:**
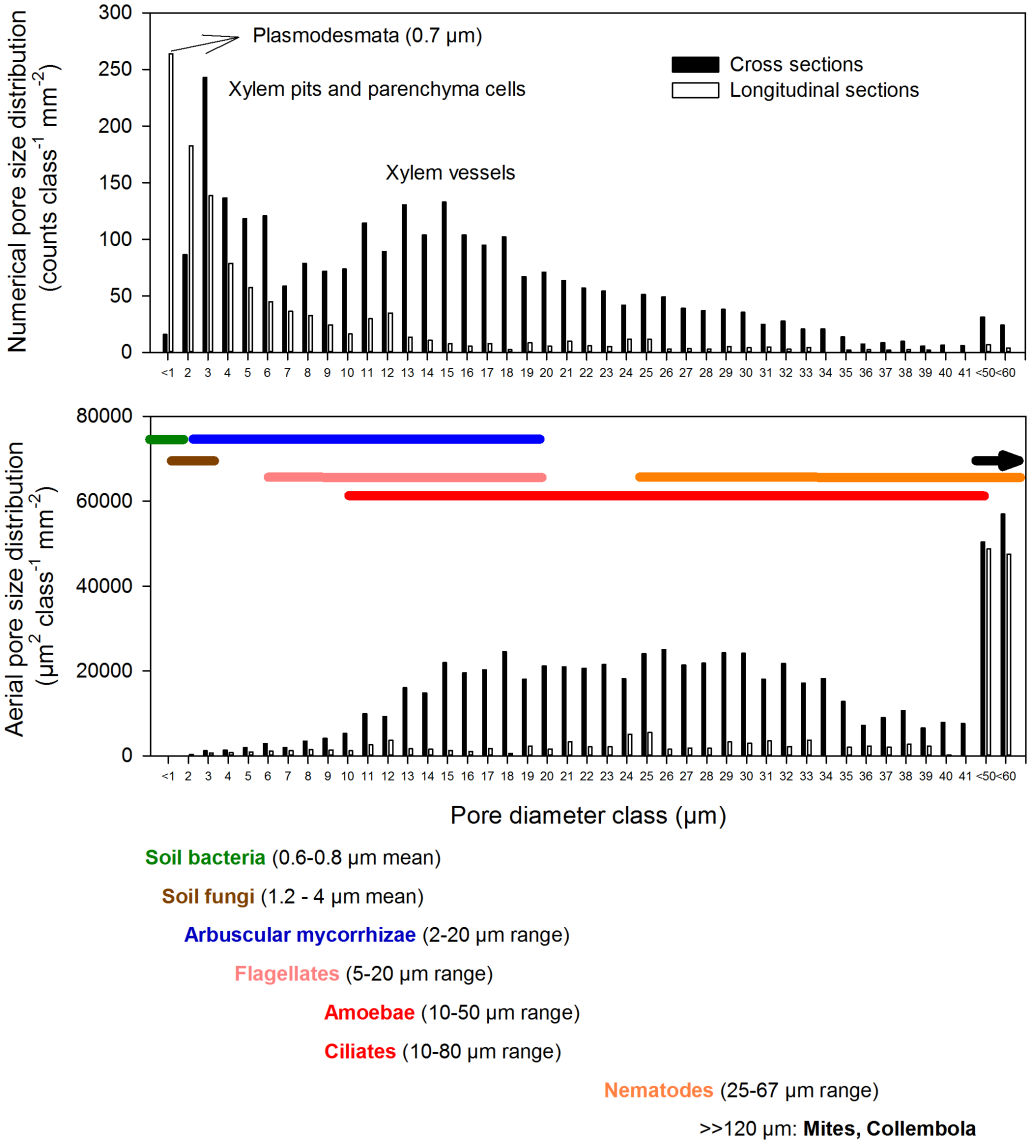
Pore size distribution of beech wood biochar as determined by scanning electron microscopy. In total, mean diameters of 7715 pores were measured in 6 longitudinal and 7 cross sections of charcoal pieces, each image covering 0.35 mm^2^. Pore size distributions are presented (1, top panel) as counts mm^−2^ per 1 µm size class (top) and (2, middle panel) on an area basis, i.e. µm^2^ mm^−2^ per 1 µm size class. Typical mean diameters (soil bacteria, soil fungi) and diameter ranges (arbuscular mycorrhizal fungi; protozoan grazers such as flagellates, amoebae, ciliates; and mesofaunal grazers and predators such as nematodes, mites and collembola) are presented color-coded (bottom panel) and with range lines in the middle panel.

The cellular structure of wood was well conserved in the biochar as seen in Figure S1. The mean pore coverage of xylem cross sections was 47.6±5.9% (mean±SD) for the biochar applied in this field trial. Overall pore size number distribution showed a maximum at approximately 0.7 µm diameter (related to simple xylem pits), and a second and third peak which are closely related to bordered xylem pits and parenchyma cells (around 3 µm) and xylem vessels (15–25 µm; Fig. 2, Fig. S2). Beech has a diffuse-porous wood structure [34] and early-wood and late-wood had distinctly different mean vessel diameters (24±12 and 15±9 µm, respectively), resulting in a decrease in mean pore coverage from 61.8±6.1% for early-wood to 40.2±4.1% for late-wood. Secondary cortex cells showed a wide range of diameters, with a peak ranging from 3 to 5 µm (data not shown). On an area basis the contribution of small pores decreased and the major contribution to total porosity shifted towards larger pores with diameters >10 µm i.e. xylem vessels (Fig. 2b).

### Effect of Biochar on General Soil Properties

The effect of biochar on soil pH and cation exchange capacity was minimal. Soil pH decreased by 0.1 units from 7.5 to 7.4 in biochar-treated soils (Table 1). Soil cation-exchange capacity decreased slightly from an initial 22.5 to 20.8 cmolc kg^−1^ in biochar-treated soils but in both cases was dominated by Ca^2+^ (∼90%) and Mg^2+^ (∼7%), with a trend towards an increase of K^+^ saturation from 1.6 to 4.8% (measured on one composite soil sample per treatment, Table 1).

**Table 1:** Soil properties in control (NPK) and biochar-treated (BC3N) soils of the Traismauer biochar field trial.

Parameter	Unit	Control (NPK)	Biochar (BC3N)
pH (CaCl_2_)		7.5	7.4
CaCO_3_	%	15.8	15.2
Humus	%	2.4	18.1
Total N	%	0.148	0.203
P (CAL)	mg kg^−1^	49	84
P_tot_ (acid digest)	g kg^−1^	5.46	5.54
Sand	%	18.3	n.d.^1^
Silt	%	57.2	n.d.
Clay	%	24.5	n.d.
CEC	cmol kg^−1^	22.5	20.8
Ca (CEC)	cmol kg^−1^	20.7	18.2
Mg (CEC)	cmol kg^−1^	1.46	1.53
K (CEC)	cmol kg^−1^	0.36	0.99
Na (CEC)	cmol kg^−1^	<0.04	<0.04
Al (CEC)	cmol kg^−1^	<0.06	<0.06
Fe (CEC)	cmol kg^−1^	<0.01	<0.01
Mn (CEC)	cmol kg^−1^	<0.01	<0.01
H (CEC)	cmol kg^−1^	0.002	0.002
Fe (EDTA)	mg kg^−1^	40	67
Mn (EDTA)	mg kg^−1^	107	128
Cu (EDTA)	mg kg^−1^	7.2	7.1
Zn (EDTA)	mg kg^−1^	2.3	7.5

All parameters were measured on composite samples collected in July 2011 from the two treatments (n = 1). n.d. – not determined.

1 Soil texture is not shown for biochar treated soils. At high contents humus biases the particle size distribution and is therefore conventionally decomposed by prolonged H_2_O_2_ (or similar oxidant) treatment prior to soil texture analysis; biochar/charcoal is largely resistant against this oxidant treatment.

### Bulk Soil C and N Contents and Soil Water Relations

**Figure 3:**
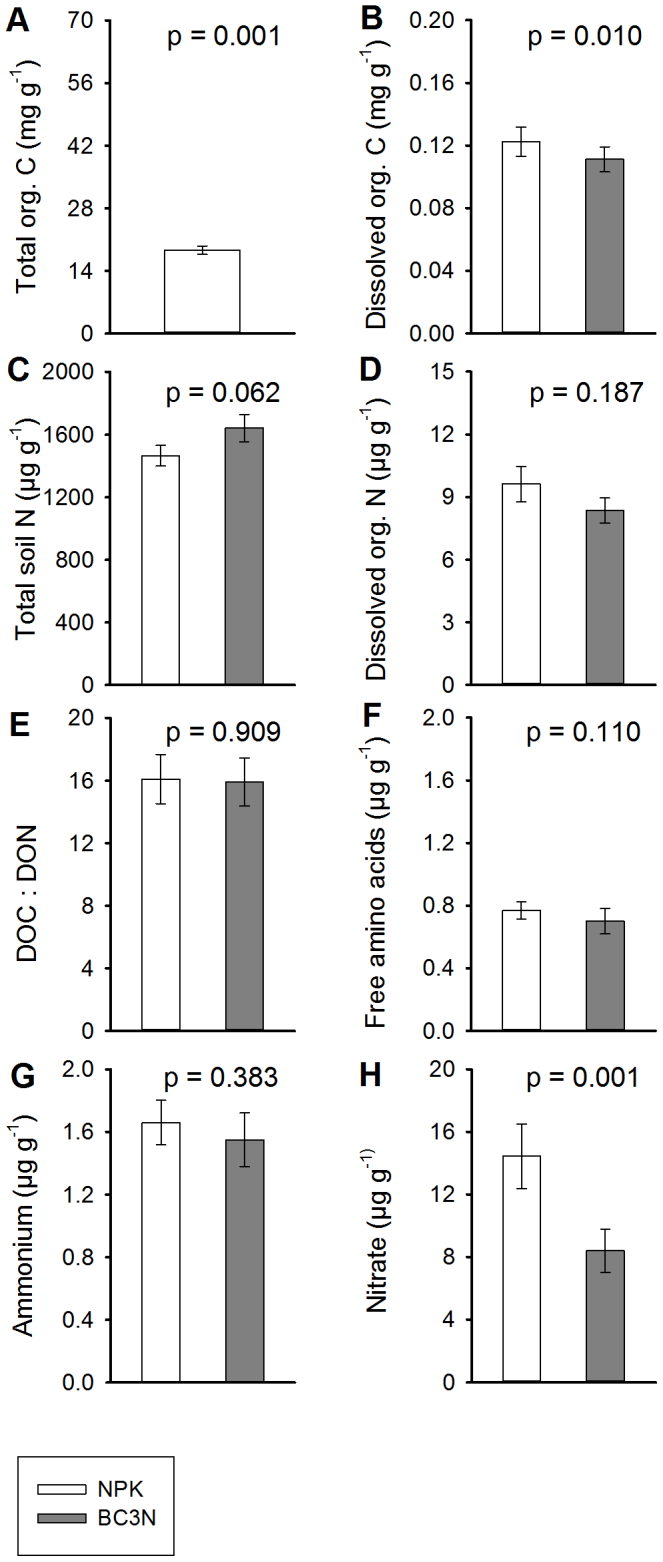
Soil C and N pool sizes for control (NPK) and biochar (BC3N) treatments. Bars represent means ±1 SE (n = 4) from seven measurements between July 2011 and September 2012 for extractable compounds, and from two measurements (September 2011 and 2012) for soil organic C and total soil N. Open bars, control treatment (NPK); grey bars, biochar treatment (BC3N). Units in mg C g^−1^ dry soil and µg N g^−1^ dry soil. P values are from two-way mixed ANOVA (see Table 2).

Biochar amendment resulted in increased soil organic C and there was a trend of enhanced total soil N concentration with biochar addition. Soil bulk densities and gravimetric soil water contents declined while water-filled pore space remained unaltered by biochar addition (two-way mixed ANOVA for effects of treatment and time, Table 2, Figure 3). Time affected most of these and the following soil parameters (Table 2), but emphasis here is given on treatment effects and treatment × time interactions; the latter might indicate a decreasing or increasing biochar-effect on soil properties or fluxes. However, all time × treatment interactions effects investigated in this study were either non-significant or not related to increasing or decreasing biochar-effects over time. Direct time effects were most likely caused by seasonal changes, agricultural management and subsequent plant growth effects. Soil organic C was significantly higher in biochar-treated soils (47.4 mg C g^−1^) than in non-biochar controls (18.7 mg C g^−1^; p = 0.001) (Table 2). Total soil N responded analogously, with a slight but non-significant increase due to biochar-treatment (1.47 to 1.64 mg N g^−1^; p = 0.062). Concurrent with increases in soil organic C the soil bulk density decreased from 1.17 to 1.04 g cm^−3^ following biochar amendment (p<0.001). Along the same lines biochar amendment caused an increase in soil porosity from 56.0 to 60.6% (p<0.001) and in gravimetric soil water content from 0.140 to 0.163 g H_2_O g^−1^ dry soil (p<0.001). As a result of increased soil water content and soil porosity with biochar addition, water-filled pore space (and air-filled pore space) did not change between controls (29.8%) and biochar-treated soils (28.9%, p = 0.229). Interestingly, by comparing all four treatments we found the biochar-effect to be amount-dependent i.e. the magnitude of the effects increased or decreased with the amount of biochar added in the field (Table S1). Soil organic C increased with biochar rate, bulk density decreased and soil porosity and soil water content increased with biochar rate (Table S1).

**Table 2:** Results of ANOVA of soil physicochemical data for the factors treatment and time in control (NPK) and biochar (BC3N) treatments.

Pools	Unit	NPK	BC3N	Treatment		Time		Interaction	
		mean	mean	F	P	F	P	F	P
C_org_	mg C g^−1^ DW	18.66	47.43	(1, 6) 43.7	0.001	(1, 6) 0.0	0.951	2.5	0.165
N_tot_	mg N g^−1^ DW	1.47	1.64	(1, 6) 5.3	0.062	(2, 12) 37.5	<0.001	0.3	0.778
BD	g cm^−3^	1.17	1.04	(1, 6) 48.4	<0.001	(1, 6) 55.3	<0.001	0.3	0.595
Grav. WC	g H_2_O g^−1^ DW	0.14	0.17	(1, 6) 46.7	<0.001	(6, 36) 218.7	<0.001	6.2	<0.001
Porosity	%	56.03	60.61	(1, 6) 48.6	<0.001	(1, 6) 55.3	<0.001	0.3	0.594
WFPS	%	29.79	28.92	(1, 6) 1.8	0.229	(6, 36) 215.4	<0.001	2.3	0.059
AFPS	%	70.21	71.08	(1, 6) 1.8	0.229	(6, 36) 215.4	<0.001	2.3	0.059
DOC	µg C g^−1^ DW	122.56	111.21	(1, 6) 13.6	0.010	(5, 30) 205	<0.001	2.9	0.028
DOC:DON		16.10	15.92	(1, 6) 0.0	0.909	(1.6, 9.9) 14.4	0.002	0.8	0.454
DON	µg N g^−1^ DW	9.62	8.35	(1, 6) 2.2	0.187	(2.3, 13.9) 28.5	<0.001	0.3	0.782
NO_3_^−^	µg N g^−1^ DW	14.43	8.40	(1, 6) 39.6	0.001	(6, 36) 95.9	<0.001	8.6	<0.001
NH_4_^+^	µg N g^−1^ DW	1.66	1.55	(1, 6) 0.9	0.383	(2.6, 15.5) 34.8	<0.001	1.0	0.417
FAA	µg N g^−1^ DW	0.77	0.70	(1, 6) 3.5	0.110	(1.6, 9.8) 3.2	0.091	0.9	0.429
TDN	µg N g^−1^ DW	24.95	17.03	(1, 6) 33.3	0.001	(5, 30) 97.2	<0.001	1.3	0.285
HMW^−^N_org_	µg N g^−1^ DW	3.24	3.47	(1, 23) 1.1	0.306				
LMW^−^N_org_	µg N g^−1^ DW	6.78	5.68	(1, 23) 0.7	0.401				
Protein	µg N g^−1^ DW	3.46	3.65	(1, 23) 1.4	0.253				
DNA	µg g^−1^ DW	12.40	18.04	(1, 6) 6.6	0.042	(1.1, 6.9) 0.1	0.774	2.6	0.154
AOA/DNA	amoA copy no µg^−1^ DNA	1.41E6	1.36E6	(1, 6) 0.2	0.637	(2, 12) 8.2	0.006	3.3	0.070
AOA/soil	amoA copy no g^−1^ soil DW	1.68E7	2.47E7	(1, 6) 6.3	0.045	(1.1, 6.6) 3.5	0.103	0.9	0.378
AOB/DNA	amoA copy no µg^−1^ DNA	419075	461583	(1, 6) 0.7	0.436	(1.2, 7) 7.2	0.028	1.6	0.246
AOB/soil	amoA copy no g^−1^ soil DW	5.01E6	8.45E6	(1, 6) 9.9	0.020	(2, 12) 4.5	0.035	1.3	0.319
AOA:AOB		3.64	3.19	(1, 6) 0.8	0.402	(2, 12) 0.4	0.681	0.0	0.970

Data were analyzed by two-way mixed ANOVA. Data for protein, HMW-N_org_ and LMW-N_org_ were available only for one sampling date and therefore were analyzed by one-way ANOVA. Abbreviations: C_org_, soil organic C; N_tot_, total soil N; BD, soil bulk density; Grav. WC, gravimetric soil water content; WFPS, water filled pore space; AFPS, aire filled pore space; DOC, dissolved organic C; DON, dissolved organic N; NO_3_^−^, nitrate; NH_4_^+^, ammonium; FAA, total free amino acids; TDN, total dissolved N; HMW-N_org_, high molecular weight organic N; LMW-N_org_, low molecular weight organic N; DNA, soil DNA content; AOA/DNA, archaeal amoA copy numbers on DNA basis; AOA/soil, archaeal amoA copy numbers on dry soil basis; AOB/DNA, bacterial amoA copy numbers on DNA basis; AOB/soil, bacterial amoA copy numbers on dry soil basis.

### Extractable C and N Concentrations

Dissolved organic C in soil extracts was slightly but significantly lower in the biochar treatment compared to the controls (111 vs. 123 µg C g^−1^ respectively; p = 0.010) (Figure 3, Table 2). Concentrations of extractable ammonium were low compared to extractable nitrate and not significantly different between the control and biochar treatments, with 1.66 and 1.55 µg NH_4_^+^-N g^−1^ respectively (p = 0.383). In contrast soil nitrate concentrations in the biochar (BC3N) treated soils were significantly lower than those from the control soils (p = 0.001), with 8.40 and 14.4 µg NO_3_^–^N g^−1^ respectively. Dissolved organic N concentrations in the soil extracts decreased, though not significantly, from 9.62 in biochar-treated soils to 8.35 µg N g^−1^ in control treatments (p = 0.187). The ratio of dissolved organic C: dissolved organic N varied around 16∶1 and was not affected by biochar addition (p = 0.909). Nitrate dominated the extractable N pool, accounting for between 67% (BC3N) and 76% (controls). The decrease in soil nitrate therefore translated into a significant decline of total dissolved N from 25.0 to 17.0 µg N g^−1^ in the biochar treatment (p = 0.001). Free amino acids were low in concentration (around 0.7 µg N g^−1^) and were not affected by treatment (p = 0.110). Measured only once (July 2011, four months after the start of the field experiment) we found no significant differences in the concentrations of different size classes of dissolved organic N between the two treatments, with extractable high-molecular weight organic N concentrations around 3.3 µg N g^−1^ and low-molecular weight organic N concentrations of around 6 µg N g^−1^ (Table 2). Free amino acids accounted for approximately 12% of the low molecular weight organic N pool and comprised 8% of dissolved organic N in both treatments. Around 40% of the dissolved organic N and all the high-molecular weight organic N fraction (>3 kDa; 105–107%) was protein, although this measurement is based only on the July 2011 sampling. The negative effect of biochar on concentrations of dissolved organic C, total dissolved N and nitrate was consistent even when all four treatments were analyzed (Table S1), with decreases in dissolved organic C, nitrate and free amino acids with increasing biochar addition rate (Table S1).

### Soil N Transformation Rates

**Figure 4:**
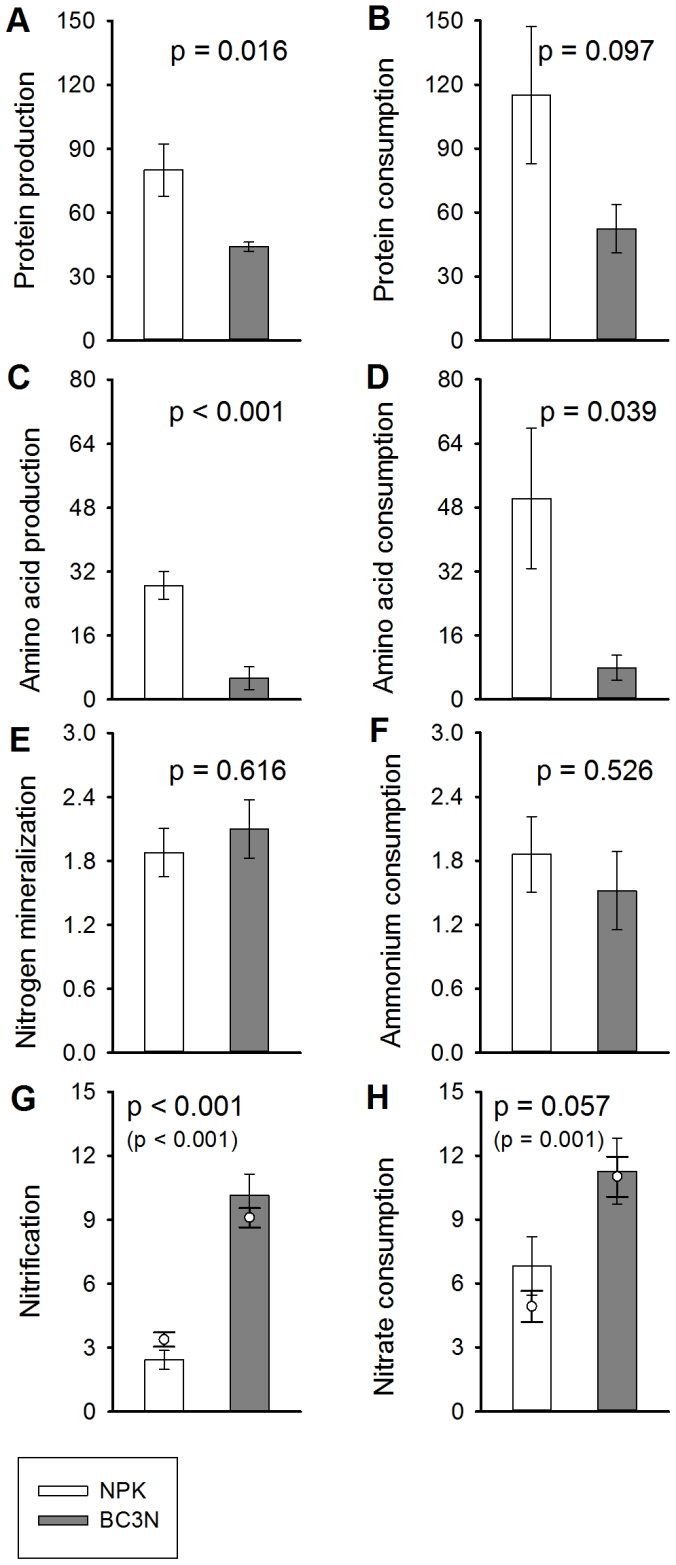
Soil N transformation rates for control (NPK) and biochar (BC3N) treatments. Bars represent means ±1 SE (n = 4) of measurements from July 2011. Open bars, control treatment (NPK); grey bars, biochar treatment (BC3N). Open circles represent means ±1 SE (n = 4) of nitrification rates from three measurement dates. Left panels represent gross influx rates into the target pools (production rates), right panels gross efflux rates (consumption rates). Units in µg N g^−1^ dry soil d^−1^, equivalent to mg N kg^−1^ d^−1^. P values are from one-way ANOVA (all rates) or two-way mixed ANOVA (nitrification for three time points, in brackets).

The full set of gross N transformation processes was measured once, and one-way ANOVA results are presented in Table 3 and Figure 4; nitrate fluxes were measured on three occasions throughout the seasons (Table 3, Figure 4, see circles). Measured rates of protein influx into the extractable organic N pool were significantly (p = 0.016) higher in the NPK treatment (80.0 µg N g^−1^ d^−1^) and almost double the rates in the biochar treatment (44.2 µg N g^−1^ d^−1^). Biochar showed a trend (p = 0.097) of depressing protein efflux rates out of the extractable organic N pool, with rates in the biochar treatment (53 µg N g^−1^ d^−1^) being half that of the control treatment (115 µg N g^−1^ d^−1^). The decline in amino acid transformation rates was even more pronounced. Amino acid influx rates into the organic N pool were significantly (p = 0.0004) lower in biochar treated soils (5.4 µg N g^−1^ d^−1^), more than fivefold lower than those in the control treatment (28.5 µg N g^−1^ d^−1^) and so too were amino acid consumption rates. Amino acid consumption rates declined more than six-fold from 50.3 µg N g^−1^ d^−1^ in the control treatment to 7.9 µg N g^−1^ d^−1^ in the biochar treatment (p = 0.039). Gross mineralization rates were not significantly different between the two treatments (p = 0.616) nor were ammonium consumption rates (p = 0.526; Figure 3), and both processes ranged between 1.5 and 2.1 µg N g^−1^ d^−1^. In contrast, gross nitrification and gross nitrate immobilization were strongly enhanced by biochar application. Gross nitrification rates in the biochar treatment (10.2 µg N g^−1^ d^−1^) were fourfold higher than the rates in the control treatment (2.4 µg N g^−1^ d^−1^), and significantly different (p<0.001). A similar trend (p = 0.0573) was evident for nitrate consumption rates which almost doubled from 6.8 to 11.3 µg N g^−1^ d^−1^ in the biochar treatment.

**Table 3:** Results of ANOVA of soil N transformation rates for the factor treatment in control (NPK) and biochar (BC3N) treatments.

Processes	Unit	NPK	BC3N	Treatment		Time		Interaction	
		mean	mean	F	P	F	P	F	P
One date									
Prot_GP	mg N kg^−1^ d^−1^	79.99	44.15	(1, 11) 8.4	0.016				
Prot_GC	mg N kg^−1^ d^−1^	115.06	52.37	(1, 11) 3.4	0.097				
AA_GP	mg N kg^−1^ d^−1^	28.54	5.36	(1, 11) 26.7	0.000				
AA_GC	mg N kg^−1^ d^−1^	50.25	7.91	(1, 11) 5.6	0.039				
N min	mg N kg^−1^ d^−1^	1.88	2.10	(1, 11) 0.3	0.616				
NH_4_^+^_GC	mg N kg^−1^ d^−1^	1.86	1.52	(1, 11) 0.4	0.526				
Nitrification	mg N kg^−1^ d^−1^	2.42	10.16	(1, 11) 50.6	<0.001				
NO_3_^−^_GC	mg N kg^−1^ d^−1^	6.83	11.27	(1, 11) 4.6	0.057				
Three dates									
Nitrification	mg N kg^−1^ d^−1^	3.38	9.09	(1, 6) 82.2	<0.001	(2, 12) 0.4	0.698	3.6	0.061
NO_3_^−^_GC	mg N kg^−1^ d^−1^	4.92	11.01	(1, 6) 35.2	0.001	(2, 12) 1.7	0.225	0.6	0.571

All transformation rates were measured and analyzed by one-way ANOVA for July 2011 data (“one date”). Nitrification and nitrate consumption was measured three times for NPK and BC3N treatments (“three dates”) and analyzed by two-way mixed ANOVA. Abbreviations: GP, gross production (influx); GC, gross consumption (efflux); Prot, protein; AA, amino acid; N min, N mineralization; NH_4_^+^, ammonium; NO_3_^−^, nitrate.

Rates of nitrification and nitrate consumption were re-measured twice. Over the course of time nitrification rates were consistently higher (threefold) in the biochar treated compared to the control plots (p<0.001, Table 3). The same pattern was apparent for nitrate consumption rates which were consistently negatively affected by biochar amendment (p = 0.001). On the sampling date where we had gross nitrification rates measured for all four treatments nitrification rates were significantly higher in all the biochar treatments (p<0.001, Table S1) with rates increasing significantly with increasing rates of biochar application.

### Microbial Community Size and Abundance of Archaeal and Bacterial Ammonia-oxidizers

**Figure 5:**
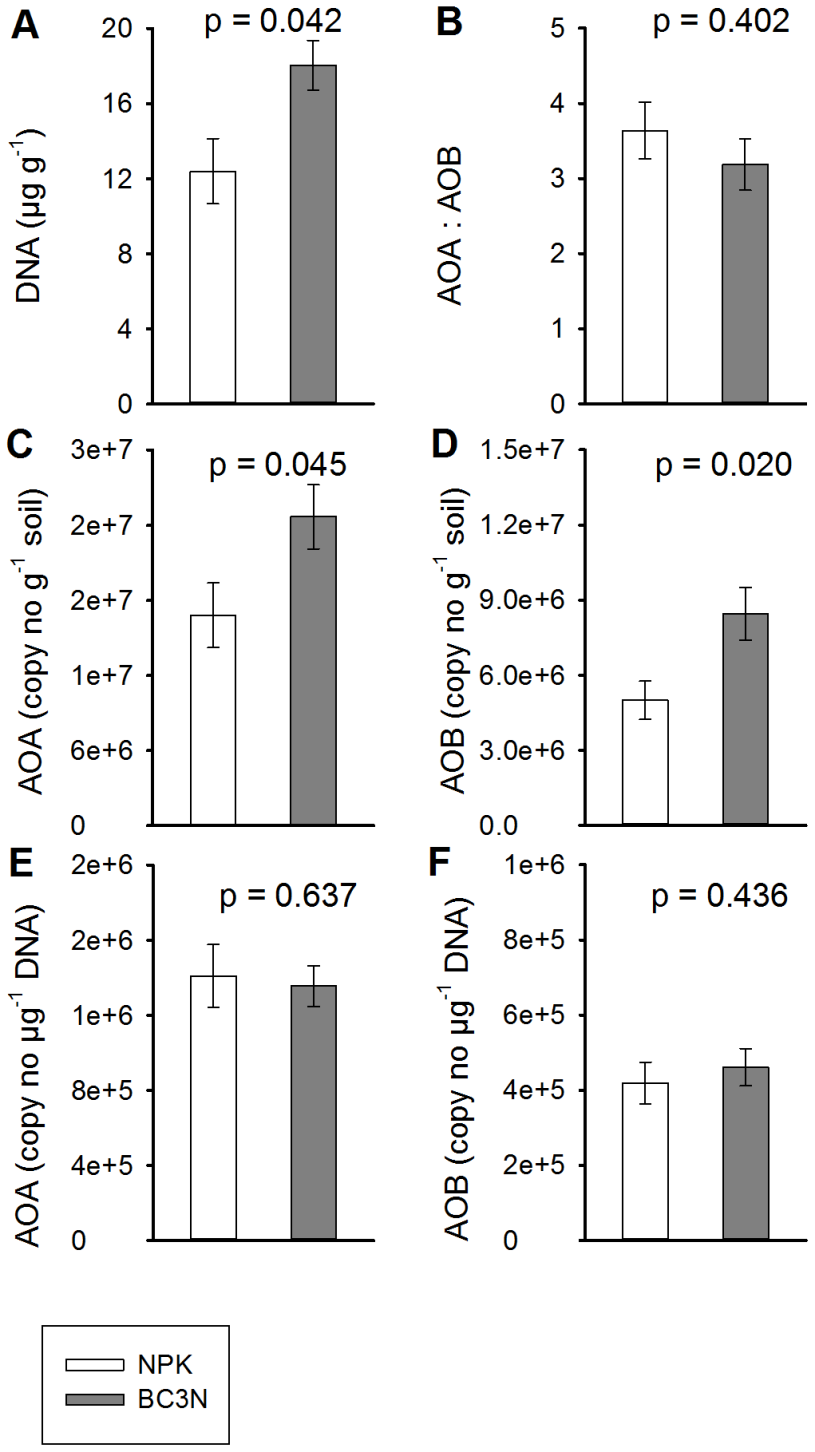
Soil microbial community size and ammonia-oxidizing community structure. Bars represents means ±1 SE (n = 4). Open bars, control treatment (NPK); grey bars, biochar treatment (BC3N). DNA, soil DNA content. AOA, archaeal amoA copy numbers; AOB, bacterial amoA copy numbers. AOA and AOB copy numbers are presented on soil dry mass and DNA basis. P values from two-way ANOVA (see Table 2).

**Figure 6:**
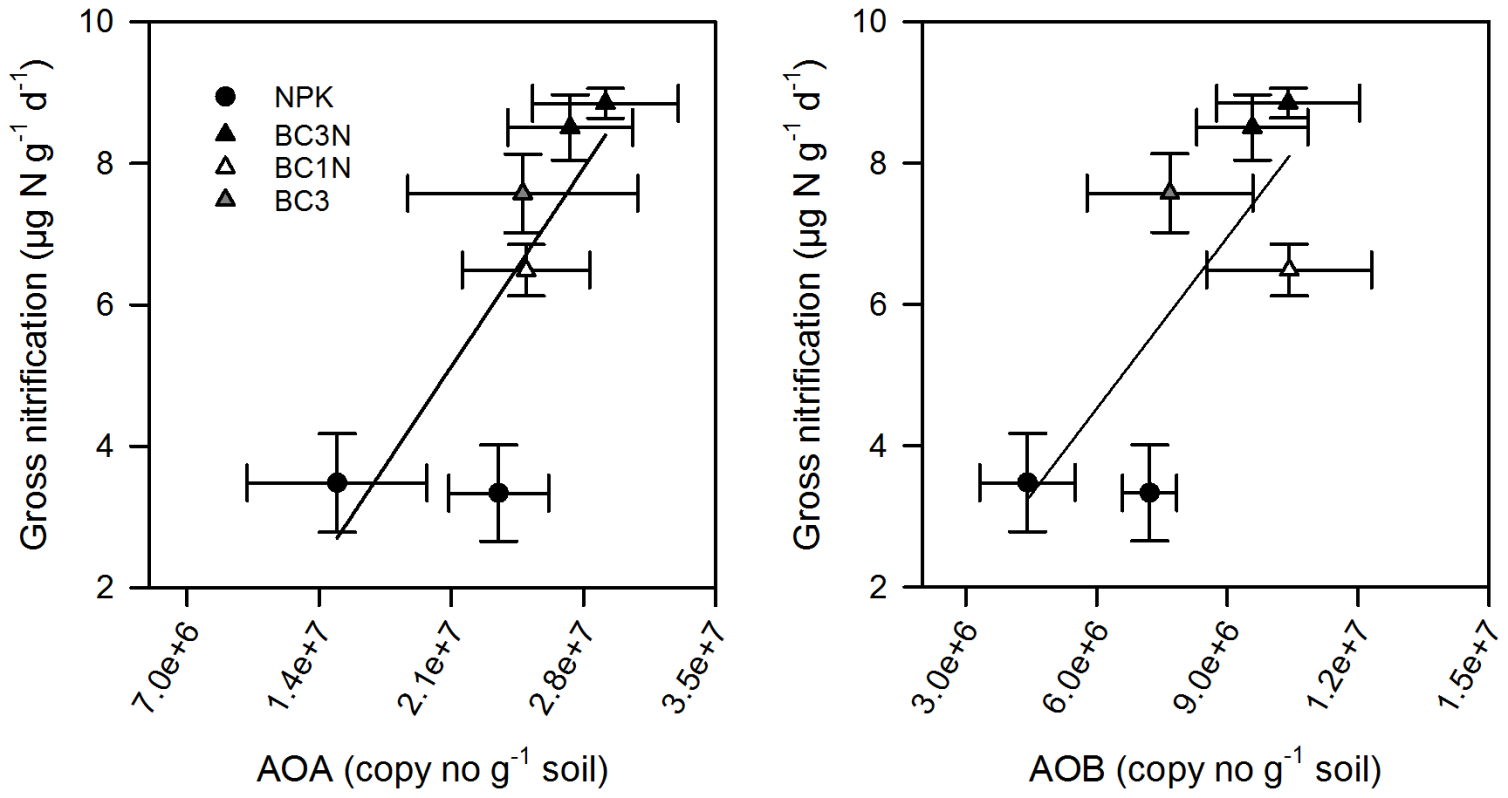
Relationship of archaeal (AOA) and bacterial amoA abundance (AOB) with gross soil nitrification rates. The relationships between archaeal amoA copy numbers (AOA) and bacterial amoA copy numbers (AOB) with gross nitrification rates were marginally significant for AOA (p = 0.051) and AOB (p = 0.072). Symbols represent the four treatments measured in September 2012. NPK and BC3N were also measured in June 2012. Values are means ±1 SE (n = 4).

Soil DNA concentration was significantly higher in the biochar treatment (18.0 µg DNA g^−1^ soil) compared to the controls (12.4 µg DNA g^−1^; p = 0.042) (Figure 5, Table 2). Ammonia-oxidizing archaea (AOA) outnumbered bacterial ammonia-oxidizers (AOB) by a factor of 3.2 to 3.6, in both treatments (p = 0.402). At the same time archaeal amoA gene copy numbers (AOA) and bacterial amoA gene copy numbers (AOB) expressed on a dry soil mass basis increased through biochar application (AOA: P = 0.045, AOB: p = 0.020). AOA increased 1.5-fold from 1.68×10^7^ to 2.47×10^7^ amoA copy numbers g^−1^ soil and AOB 1.7-fold from 5.01×10^6^ to 8.45×10^6^ amoA copy numbers g^−1^ soil through biochar amendment. As a result of increasing soil DNA concentration and amoA copy numbers per gram soil we did not find a significant effect of biochar on the abundance of AOA (p = 0.637) and AOB (p = 0.436) expressed on a DNA basis, i.e. the relative share of ammonia oxidizers in the microbial community did not change according to treatment. Across all four treatments we did not find an effect of biochar rate on either soil DNA concentration or any measure of AOA or AOB abundance (Table S1). However, archaeal and bacterial amoA gene copy numbers were strongly positively related (R^2^ = 0.83, p = 0.0114; data not shown). Moreover, we found a trend towards a positive relationship between both, AOA or AOB numbers on a dry soil mass basis and gross nitrification rates (AOA: R^2^ = 0.65, p = 0.051, AOB: R^2^ = 0.60, p = 0.072; Figure 6). The “weakness” of this relationship derived mainly from the control treatment where nitrification rates were relatively constant between the two measurement dates while amoA copy numbers varied more strongly. Nitrification rates were significantly positively correlated with the total number of amoA copy numbers on a soil dry mass basis (AOA plus AOB; R^2^ = 0.66, p = 0.049, data not shown).

## Discussion

### Soil Organic N Cycling

Biochar addition had a strong negative effect on soil organic N cycling rates as it reduced protein production and protein consumption and concurrently decreased free amino acid production and consumption (Figure 4). This is important given that protein depolymerization plays the predominant role in organic N recycling with approximately 50% of soil organic matter and >90% of plant detritus being composed of protein [35]–[36], and with proteins representing around 40% of dissolved organic N in this study. Proteins are therefore considered the most important substrate for microbial N mobilization and N use [33], and thus any change in their dynamics strongly impacts whole-system N cycling. The influx into the K_2_SO_4_ extractable protein pool (representing a labile part of the total protein pool in soils) is mainly fuelled by microbial turnover and protein desorption processes while efflux from this pool relates to protein depolymerization or protein adsorption (Figure 1).The concomitant reductions in free amino acid turnover were most likely a direct consequence of the reduced protein-N cycling in biochar amended soils. Amino acid production is driven by depolymerization of accessible proteins through extracellular proteases and peptidases which release oligopeptides and free amino acids that are then taken up by microbes (free amino acid consumption) [33]. Though oligopeptides also represent a significant source of N for microbes and plants [37] we did not measure their dynamics, which would have been expected to change accordingly. We offer two possible explanations for the observed negative effects of biochar on organic N (protein) dynamics related to the surface properties of biochar and its unique pore structure.

1. The observed decline in protein production rates might result from protein adsorption to biochar surfaces. The reactive surface of biochar has been shown to adsorb numerous low and high molecular weight organic molecules [38], including proteins and enzymes [39]. Hence, the reduced motility and accessibility of proteins in the biochar treated soils could explain the decrease in protein influx rates observed with resultant implications for amino acid dynamics. Very strong H-bonding between proteins and polyphenols are well known [40], and the abundant phenolic groups on low temperature biochars [41] provide ample ligands for strong sorption of proteins and peptides [9].
2. Biochar could also provide structural protection, by the entrapment of organic matter within its inherent pore network. The “mesopore protection hypothesis” by Zimmerman et al. [42] suggests that small organic molecules can be occluded within mineral mesopores (2–50 nm diameter), physically protecting organic molecules such as proteins (<10 nm) and amino acids (<2 nm) from breakdown by excluding decomposers and their extracellular enzymes. Mesopore surface area and volume are mainly determined by gas sorption analysis but are not targeted by scanning electron microscopy, and therefore have not been measured in this study. Nevertheless, low-temperature wood biochars produced at <650°C, just as the Romchar used in this study, have been shown to have a low mesopore volume and surface area as measured by gas sorption [39]. Physical protection of organic matter in mesopores by occlusion from decomposing microbes may thus have played a minor role in this study.

Just like the decrease in protein production, the decline in free amino acid production in biochar treated soils could also be triggered by protease and peptidase adsorption to or occlusion in biochar. Enzyme adsorption to solid support materials (e.g. soil matrix or biochar) has been reported to decrease soil enzymatic activity [43], and adsorbed substrates such as proteins and oligopeptides are also less accessible and bioavailable for further breakdown to free amino acids. Decreased aminopeptidase activity has been observed in experiments with fast pyrolysis biochar of switchgrass and was ascribed to sorption reactions between substrate and biochar [44]. Another study found accelerated N-mineralization of the recalcitrant soil organic N pool in arable soils treated with maize biochar [14]. The inconsistent effects of biochar on soil enzymes may be the result of several factors such as biochar feedstock, pyrolysis conditions, soil type as well as the respective enzyme and/or substrate [44]. For example, contrasting results obtained in our experiment and that of Nelissen et al. (2012) [14] could be explained by structural differences between the biochars used in these two studies, leading to variable enzyme diffusion impedance. Indeed, the Romchar employed here maintained its native wood-like structure according to the scanning electron microscopy images (Figure S1) while non-woody materials such as maize biochar have an amorphous structure with little evidence of retained pores [45].

### Soil N Mineralization

Given that biochar application significantly decelerated the production and consumption of low molecular weight organic N in our field experiment, we would have expected a decrease in soil N mineralization rates. Somewhat surprisingly biochar had no significant effect on gross N mineralization and gross ammonium immobilization rates in our study, in line with a laboratory incubation of cultivated black Chernozem soil with wheat biochar [46], but in contrast to observed accelerated rates of gross N mineralization of the recalcitrant soil organic N pool in an arable soil treated with maize biochar [14]. Interestingly mineralization (respiratory use) of amino acids and peptides, measured as the uptake of ^14^C-labelled organic N and subsequent ^14^CO_2_ release, was not affected by application of a wood-based biochar [15]. Divergent, positive, neutral or negative effects of biochar on soil N mineralization may be the upshot of different biochar types along with impacts on the C and N status of the soil microbial community. Carbon-rich biochar, poor in nitrogen, such as beech wood biochar (C:N ratio ∼200∶1) might promote N limitation, leading to the retention of catabolically produced ammonium in the N-limited microflora, which therefore results in a decrease of the amount of ammonium released to the soil [29]. Conversely, nitrogen-rich biochars with low C:N ratios such as maize-biochars [14] or manure-biochars [47] promote microbial C limitation, causing the excess of N to be mineralized and therefore N mineralization rates to increase. In our case the ammonium released (N mineralization) relative to organic N taken up by microbes (free amino acid consumption) was higher in the biochar treated soils than in controls (Figure 4), indicating that biochar addition did not lead to microbial N limitation as a consequence of the increased organic C content in soil. This suggests that the majority of organic C in the biochar was not metabolized or assimilated by the soil microbial community, and confirms that biochar is an efficient tool for long-term C sequestration in soil, with half-lifes >100 years [48]. The absence of microbial N limitation in the biochar treatment might be directly related to the field applications of fertilizer and might also be related to the slightly lower concentrations of dissolved organic C in the biochar treated plots.

### Soil Nitrification

Apart from the negative effect that biochar had on soil organic N cycling, the most striking result was a strong and persistent (1.5 years) stimulation of gross nitrification. Several studies have examined the impact of biochar on soil net nitrification [8] but only few have reported gross nitrification rates, which allow the simultaneous analysis of both, productive and consumptive processes for nitrate. Biochar addition increased gross nitrification rates in two forest soils and one arable soil [14], [26], [49] but had no effect in other experiments with grassland and arable soils [46], [49]. Several hypotheses have been raised to explain biochar-related (positive) effects on nitrification rates.

One of the dominant theories to explain the increase in nitrification rates following biochar amendment is the liming effect [14], [26]. Biochar commonly has a neutral to alkaline pH [50] and therefore may increase the pH of acidic soils with a concurrent positive response of soil nitrifiers whose pH optimum is slightly acidic to neutral pH [51]–[52]. However the pH of the soil used in this study was slightly alkaline and did not increase but rather decreased in response to the addition of biochar.

Equally we can disregard the tenet that biochar addition leads to an increase in nitrification due to adsorption of nitrification inhibitors. In contrast to forest and grassland soils [49], [53]–[54], intensively managed agricultural soils usually contain low amounts of naturally occurring nitrification inhibitors [55] and their effects might have been of minor importance in this experiment.

Nitrification rates have been shown to be strongly controlled by the ammonium supplied by soil N mineralization in a wide variety of ecosystem types including woodland, grassland and agricultural soils [56]. In this study, biochar amendment neither had an effect on gross ammonium production rates nor on the pool size of extractable ammonium, which obviates a simple substrate-driven explanation of increased nitrification rates in this field experiment. Whereas nitrification rates in the control soils were of the same order of magnitude as the mineralization rates, biochar treated soils exhibited nitrification rates four to five times higher than mineralization rates. This dichotomy between ammonium supply and nitrification rates could point to increased importance of heterotrophic nitrification fuelled by organic N compounds in biochar-treated soils. However heterotrophic nitrification mainly occurs in grassland and forest soils and especially in acidic soils [57]–[58], although this process has been recently detected in acidic arable and neutral soils [59]–[60]. This nitrification pathway will have to be investigated in follow-up incubation experiments, using acetylene as an inhibitor of autotrophic (archaeal and bacterial) nitrification [61] which does not affect heterotrophic nitrification [62]–[63].

Increased nitrification rates in soils treated with biochar could be attributed to the alteration of the soil water status, including its distribution and associated changes in soil oxygenation, since nitrification is dependent on soil O_2_ availability. The soil air/water balance is essential for nitrification, which has been found to peak at 60% water-filled pore space [64]. Altered soil moisture conditions have been reported in various biochar studies [17], [65]–[66]. However, in this study air-filled pore space was highly variable and on average greater than 70%, independent of treatments; therefore higher nitrification rates (Figure 4) could not be explained by improved oxygen availability.

While none of the proposed drivers of soil nitrification could explain the observed pattern of enhanced nitrification in biochar treated soils, the size of the ammonia-oxidizing community increased with biochar amendment (Fig. 5). Recently, the oxidation of ammonia to nitrite, the first and rate limiting step of nitrification, was shown to be performed by prokaryotes in both, the bacterial and archaeal domains [67]. Both ammonia oxidizing archaea (AOA) and ammonia oxidizing bacteria (AOB) abundances were significantly higher in the biochar treated soils, AOA outnumbering AOB in both treatments. There are numerous studies showing that AOA are often significantly more abundant than AOB in soils of different origins [68]–[69]. However the relative importance and contribution of these different groups to the nitrification process in natural environments is still unclear and to our knowledge there are no published studies available on the interaction of biochar and AOA, at least in temperate ecosystems. AOB abundances have been found to increase after natural forest fire events most likely as a consequence of the liming effect of charcoal and its sorption of nitrification inhibitors [26]. In a pot experiment the AOB community structure was unaffected by the sole addition of *Eucalyptus* wood biochar but changed when biochar was added together with an inorganic or organic N source [70], a possible result of substrate-limited nitrification as the ammonium concentrations declined in sole biochar treatments. The stimulation of both, AOA and AOB abundances through biochar addition in this field experiment was only significant when amoA gene copy numbers were expressed on a soil mass basis but not when expressed on a soil DNA basis. The microbial community size, indicated by total soil DNA content, increased with biochar addition revealing a general positive effect of biochar on the whole microbial community rather than a specific stimulation of the autotrophic nitrifiers. This unspecific stimulation of microbes and concomitant increase of nitrifiers is corroborated by the lack of biochar effects on more specific soil nitrification controls such as soil pH, ammonia and oxygen availability, and presence of nitrification inhibitors. The biochar effect was also unspecific in terms of stimulation of AOA and AOB; both AOA and AOB abundances increased with biochar and were strongly positively related across all soil samples independent of treatment. In addition ratios of AOA: AOB were unaffected by the biochar amendment.

AOA and AOB abundances were broadly (P<0.1) correlated with gross nitrification rates, suggesting that bacterial and archaeal abundance increased with increasing biochar addition and providing a functional link to the process data (Fig. 6). This and the total DNA data add weight to an underlying hypothesis, i.e. that the structural nature of the biochar provides a protective habitat for bacterial and archaeal growth. It is conceivable, that biochar promotes bacterial and archaeal community size by enhancing the colonizable surfaces in the soil and providing habitable protected pores. The unique pore-size distribution typically found in biochar with pores ranging from 1 nm to 100 µm [71] is believed to provide various beneficial functions to soil bacteria and archaea [72]. Soil prokaryotes typically have mean cell sizes of 0.6 to 0.8 µm in diameter [73]–[74] with a length: diameter ratio of 1.5∶1 [75] and were shown to inhabit pores greater than 0.6 µm [76]. Soil fungi have slightly larger mean diameters, of between 1.2 to 4 µm [74], [77] but mycorrhizal hyphae vary enormously in diameter ranging between 2 to 20 µm [78]. As a consequence of the high proportion of colonizable pores, addition of *Eucalyptus* charcoal to a coarse textured soil increased habitable pore space surface area by 11% at a biochar rate of 25 t ha^−1^ [70] and habitable pore volume by 6.5% at 50 t ha^−1^ [79]. Indeed we found an increase of pore volume of 8% at biochar rates of 72 t ha^−1^. More importantly it has been shown that interior surfaces and pores are rapidly colonized, microbiota showing no differences in densities between outer and internal biochar surfaces 3 years after application [79]. It has been suggested that soil microbes are protected from being grazed upon their natural predators by colonizing pore habitats in biochar that are inaccessible for soil microfauna (Fig. 2). By this mechanism biochar pores can act as a safe microhabitat for various soil microbes through the exclusion of larger predators [80]–[81] such as nematodes, mites and collembolans [73] but also microfaunal protozoan grazers and predators, i.e. flagellates, amoebae and ciliates [82]–[83]. Our scanning electron microscopy data suggest that there was an abundance of colonizable pores, particularly in the 0.3 to 40 µm range with maxima around 0.7, 3 and 15 µm. The abundant sack like structures of parenchyma cells and the long cave like structures of xylem vessels (Figure S1) could therefore offer physical protection for soil prokaryotes and fungi from grazers such as nematodes and amoeba, which are just physically too big to enter the cave or pore. This in turn could reduce microfaunal grazing, which leads to a decrease in mineralization of organic N via the soil “microbial loop” [84]. This scenario provides a possible explanation for the observed increases in nitrification rate, with bacteria, fungi, AOA and AOB taking refuge in the biochar. In the biochar pores AOA and AOB have ample access to carbon dioxide and available ammonia, given the high pH of this soil; this allows them to proliferate, but protected from grazing, thus not contributing significantly to N mineralization. Interestingly this scenario would suggest possible top down control of nitrification, with predators having been shown to consume up to 60% of bacterial production in some soils [85]. In addition to the provision of protected habitat provided by biochar co-location of nutrients and water on biochar surfaces and in pores may increase substrate availability, and thus may further stimulate microbial colonization [72] and nitrification.

## Conclusions and Outlook

The main findings of this field study are that application of wood biochar led to marked decreases in soil organic N transformation rates while soil nitrification was significantly stimulated in this type of agricultural soil. This field study therefore indicates a de-coupling of the intrinsic organic and inorganic soil N cycles in the short- to medium-term (four to 18 months) as a consequence of biochar addition. Assuming these effects persist, the deceleration of soil organic N cycling ultimately leads to the dichotomy of two intertwined agronomic processes; the build-up of soil organic N and the slowing-down of inorganic N release from soil organic N essential for crop growth. Addition of fertilizer-N in combination with biochar effectively decouples these processes, with plants and microbes utilizing fertilizer-N for growth, in turn fuelling the belowground build-up of soil organic N. Since soil organic N and particularly refractory soil organic matter are peptide dominated materials [86], the accumulation of soil organic N is expected to enhance soil carbon sequestration due to increases in non-biochar derived soil organic matter. However, it remains an open question whether adsorptive capacity of biochar will saturate and pore-filling of biochar slows or curtails this process, though recent investigations with aged and fresh biochar suggested that the effects of biochar on organic compound sorption may be long lasting [87].

Stimulation of soil nitrification can have positive effects on plant N availability and crop growth but concurrently increases the risk of soil N losses through nitrate leaching and gaseous losses by denitrification and nitrifier-denitrification. The decrease in soil nitrate concentration and increase in microbial nitrate utilization however point to a relatively conservative N cycling in biochar-treated arable soils. It will therefore be of great importance to study the responses of soil organic N, nitrification, soil N losses and plant growth over longer time periods and in various agricultural settings, to better understand the long-term effects of biochar amendments on soil N cycling.

Given the central role of soil organic N cycling and nitrification in controlling global terrestrial N cycles, the implications of this work are of major importance. If it is more broadly proven that addition of biochar combined with fertilizer-N leads to a greater than sum increase in non-biochar derived soil organic matter, as a consequence of the mechanisms described here, it may prove to be a fundamental factor in future soil management: as building soil organic matter through carbon farming is the key step required to kick start the next black/green revolution.

## Materials and Methods

### Description of Field Trial

The field experiment was established in Traismauer, Lower Austria, Austria (48° 19′ 886′′ N, 15° 44′ 308′′ E; 268 m a. s. l) in March 2011 by the Austrian Institute of Technology (AIT) Tulln, Austria. The temperate climate shows strong continental influence (Pannonian climate), with mean annual precipitation of 550 mm and mean annual temperature being 10°C. The arable soil was a deep sandy to loamy silt (18.3% sand, 57.2% silt and 24.5% clay), classified as a calcareous Chernozem on loess. Soil pH of non-biochar treated soil was 7.5 in 10 mM CaCl_2_, the carbonate content was 15.8% and humus content 2.4% (Table 1). Cation exchange capacity of the soil was 22.5 cmol kg^−1^, dominated by Ca^2+^ (∼90%) and Mg^2+^ (∼7%), with a small contribution by K^+^ (2%). The site was used for agriculture for >100 years, with spring barley grown in 2011 and sunflower in 2012. Hardwood-derived biochar (80% beech, rest derived from other hardwoods) was produced by pyrolysis at 500°C for two hours and was purchased from S.C. Romchar S.R.L. (Harghita, Romania). This biochar had a pH of 8.6 in water and 7.5 in 10 mM CaCl_2_ and comprised the following elemental concentrations: 80.3% C, 9.9% O, 0.4% N, and 1.6%H contents (Table 4). Volatiles made up 11.1% and ashes 15.2% of biochar dry mass (both determined at 550°C, Table 4). Biochar was applied once to the field at a dry mass rate of 0, 24 and 72 t ha^−1^ in March 2011 and incorporated to a depth of 15 cm. NPK fertilizer was applied annually in spring providing an annual N input of 120 (2011) and 75 kg ha^−1^ (2012), with corresponding annual rates of P input of 26 (2011) and 31 kg ha^−1^ yr^−1^ (2012) and K input of 50 (2011) and 100 kg ha^−1^ yr^−1^ (2012). The four treatments were: control i.e. NPK (no biochar application, NPK added), BC1N (24 t biochar ha^−1^, NPK added), BC3N (72 t biochar ha^−1^, NPK added) and BC3 (72 t biochar ha^−1^, no NPK added). The four treatments were arranged according to a Latin square and replicated four times in circular plots of 6.5 m diameter.

**Table 4:** Element contents of beech wood biochar pyrolyzed for two hours at 500°C.

Element	Content (% d.w.)
C	80.30
O	9.88
Ca	3.75
H	1.60
Si	1.58
Fe	0.79
K	0.73
N	0.40
Al	0.243
Mg	0.198
Mn	0.091
P	0.076
Si	0.053
Cl	0.010
Zn	0.009
Cr	0.005
Co	0.002
Ashes (550°C)	15.2
Volatiles (550°C)	11.1

The field is privately owned and not protected in any way. The local winegrower association Traisen valley (Weinbauverband Traisental) gave us the written permission to conduct the field experiment at the premises owned by Rudolf Hofmann who also agreed in his role as landowner to set up the experimental plots on his land. No governmental permits were required for the described field study, and the study did not involve endangered or protected species.

Soil sampling was performed several times in 2011 and 2012 (see Table S2 for sampling dates and for the treatments sampled and the pools and processes measured at each date). Soil samples were taken from the topsoil layer (0–10 cm) using a soil corer with 8 cm diameter. From each treatment plot three soil samples were collected randomly and pooled to form one composite sample. The fresh samples were then weighed for bulk density calculation, sieved to pass 2 mm and stored at 4°C for a maximum of 2–3 days prior to analyses.

### Soil Physicochemical Analyses

#### General soil properties

Soil water content (SWC, in g H_2_O g^−1^ dry soil) was determined gravimetrically after drying fresh soil at 105°C for 24 hours in a forced-air drying oven. Bulk density (BD, in g cm^−3^) was calculated from the fresh weight divided by soil core volume times dry weight: fresh weight ratio. The percentage of water-filled pore space (%WFPS) was calculated as follows:
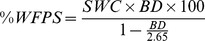
where 2.65 g cm^−3^ represents the mean density of rock.

Soil total C and N contents were measured on oven-dried (105°C) and homogenized soil samples. Samples were ground to a fine powder with a ball mill (MM2000, Retsch, Germany) and aliquots of 10 mg weighed into tin capsules. Total soil C and N contents were determined by an elemental analyzer (EA 1110; CE Instruments, Italy). For quantification of the soil organic carbon content samples were pretreated with excess acid (2 M HCl) for 60 min and then dried at 105°C to remove carbonate from the calcareous soil. Other basic soil properties, such as soil pH, soil organic matter content, carbonate content, soil texture, total P content, cation-exchange capacity, base saturation, and exchangeable cations (see Table 1) were only measured for control (NPK) and biochar-treated soils (BC3N) at the Institute of Sustainable Plant Production, Federal Agency for Food Security (AGES) in Vienna, Austria, according to standard ÖNORM procedures (www.austrian-standards.at/en).

#### Biochar characterization

The biochar (beech wood charcoal) was characterized for element, volatiles and ash contents by the German Biomass Research Center (DBFZ, Leipzig, Germany) and results are presented in Table 4. Biochar pH was measured in an aqueous slurry (5% (w/v)) using a pH electrode. For measurements of the porosity (pores >300 nm diameter) and the pore size distribution of the biochar, charcoal samples were mounted on 0.5″ aluminum specimen stubs equipped with SEM-Carbon foils (Agar scientific, UK). Since the specimens were conductive no additional coating was performed after air drying at 40°C for 24 hours. Micrographs were taken on a Philips XL 20 scanning electron microscope (SEM; Philips, Eindhoven, The Netherlands) at 350× and 2000× magnification with an acceleration voltage of 15 kV. For pore size and pore coverage measurements the software Scandium Version 5.0 (Soft Imaging Systems GmbH, Olympus, Vienna, Austria) and the image analyzing software GSA Image Analyzer (GSA, Gesellschaft für Softwareentwicklung und Analytik mbH, Germany) were used. To estimate pore size distribution in total, 7715 pore measurements in 6 longitudinal and in 7 cross-sectional images covering 0.35 mm^2^ and in 25 longitudinal images covering 0.0112 mm^2^ were performed. Pores down to a diameter of 300 nm could be safely measured. Pore size distributions were converted to counts mm^−2^ or circular area in µm^2^ mm^−2^ and summed up for each 1 µm size class.

#### Pool sizes of extractable organic C, inorganic N and organic N forms

For measurements of extractable C and N pools fresh soils (aliquots of 4 g) were extracted for 30 min with 30 ml 0.5 M K_2_SO_4_, subsequently filtered through ash-free cellulose filters and extracts kept frozen at −20°C for later analyses. Total dissolved N and dissolved organic carbon (measured as non-purgeable organic carbon) were measured by high temperature catalytic oxidation on a Shimadzu TOC-VCPH with TNM-1 unit and ASI-V autosampler (Shimadzu Austria, Korneuburg, Austria). Ammonium and nitrate were determined with colorimetric methods, NH_4_^+^ with a modified indophenol reaction and NO_3_^−^ with the VCl_3_/Griess assay, as described in detail by Hood et al. [88]. Amino acid concentrations were quantified by a modified fluorometric OPAME procedure based on Jones et al. [89], optimized for free amino acid measurement in protein hydrolysates. The modified OPAME reagent was made up of 10 mg o-phthaldialdehyde dissolved in 1 ml of HPLC grade methanol to which 20 µl of 3-mercaptopropionic acid was added. This reagent was mixed with 40 ml potassium borate buffer (0.2 M; pH 9.5) and left to stand overnight. Sample aliquots (50 µl) were mixed with 200 µl reagent in the microtiter plate and measured after 10 min at an excitation wavelength of 340 nm and emission wavelength of 450 nm with a fluorescence microplate reader (Tecan Infinite M200, Tecan Austria GmbH, Grödig, Austria). As NH_4_^+^ in the soil extracts also gives a fluorescence signal, NH_4_^+^ concentration had to be determined [88] and its fluorescence contribution was determined via a NH_4_^+^ concentration series and subtracted from the total fluorescence of the samples. Total free amino acid-N concentration was then calculated using glycine as a standard and assuming that each mol of amino acid contains 1.37 mol N. Proteins were quantified in K_2_SO_4_ extracts by acid hydrolysis and subsequent amino acid analysis (modification of [89]–[90]; see amino acid determination above). Protein hydrolysis to free amino acids was performed in 6 M HCl and heating was performed for 15 hours at 100°C in a drying oven with Bovine serum albumin (BSA containing 15.8% N) as internal standard. Samples were then dried in an N_2_ stream and redissolved in deionized water. Determination of the NH_4_^+^ concentration to correct the amino acid fluorescence was done as in the case of amino acid determination, with the exception that the sodium nitroprusside solution (the color reagent) contained 1.5 M NaOH instead of the original 0.3 M NaOH to neutralize residual acid in the hydrolysates.

### Isotope Pool Dilution Assays

Gross rates of N transformation processes were determined using the ^15^N isotope pool dilution (IPD) technique [31], [91]. This method entails the addition of an isotopic tracer to a target pool and measuring the isotopic composition and concentration over time which allows estimating gross rates of influx to (production) and efflux from (consumption, immobilization) the target pool. In order to avoid the stimulation of consumption rates due to excessive addition of ^15^N labeled substrate to the respective target pool, each pool size was determined prior to labeling the soil samples. Thereafter to each target pool 10% of the pool size was added in the form of ^15^N tracer (98 at% ^15^N). Before starting the IPD assays, all soils were adjusted to 40% water holding capacity and then the ^15^N tracer solutions were added to bring the soils to 70% water holding capacity. After addition of the tracer to the soils the vials were shaken vigorously to homogenously distribute the label. The incubations were performed at room temperature (∼20°C) and terminated by adding 0.5 M K_2_SO_4_ and subsequent extraction for 30 min on a rotary shaker followed by filtration through ash-free filter papers into new vials. Extracts were immediately frozen and kept for further analyses. An overview of the soil preparation pipeline for these isotope pool dilution assays is presented in Figure S2.

#### Gross N mineralization and NH_4_^+^ immobilization

Each 2 g soil sub sample was weighed into disposable HDPE vials (20 ml scintillation vials) and labeled with ^15^NH_4_Cl (98 at% ^15^N purchased from Isotec-Sigma Aldrich, Vienna, Austria). The incubations were terminated after 4, 24 and 48 hours by extraction with 15 ml 0.5 M K_2_SO_4_. Prior to isotopic analyses ammonium concentrations in the labeled extracts were measured photometrically. Ammonium was isolated from the extracts by microdiffusion [92]. In brief, this method implies the conversion of ammonium into ammonia gas which is then trapped on an acidified cellulose filter disc enclosed in a semi-permeable Teflon membrane (acid trap), where the ammonia gas dissolves and redissociates to ammonium. The interconversion of ammonium and ammonia is a consequence of the pH-dependent equilibrium between these two N forms. To each 10 ml of soil extract one acid trap and 100 mg MgO were added to raise the pH to >9.5, the vials were closed immediately and left to stand at room temperature for three days. In cases where ammonium concentrations exceeded the optimal range of N (10–25 µg NH_4_^+^-N 10 ml^−1^) for elemental analyzer-isotope ratio mass spectrometry (EA-IRMS) measurement, extracts were diluted with 0.5 M K_2_SO_4_ prior to microdiffusion. After microdiffusion acid traps were transferred to 2 ml HDPE screw-cap vials and dried in an evacuated desiccator over concentrated sulphuric acid for two days. The cellulose discs were retrieved and transferred into tin capsules and subjected to EA-IRMS. Conversely extracts with ammonium-N concentrations less than 2 µg NH_4_^+^-N 10 ml^−1^ were prepared to be measured with purge-and-trap isotope ratio mass spectrometry (PT-IRMS) [93]. For this purpose acid traps were made from glass fiber filters. Acid traps to be measured with PT-IRMS were opened and the filter discs transferred to 1.5 ml HDPE snap-cap vials containing 1 ml deionized water and shaken on a Vortex to redissolve the trapped ammonium. The alkaline persulfate oxidation with subsequent VCl_3_/azide reactions were then applied to convert NH_4_^+^ via NO_3_^−^ to N_2_O for analysis by PT-IRMS (see below) [93].

#### Gross nitrification and NO_3_^−^ immobilization

Soil labeling was done with K^15^NO_3_ (98 at% ^15^N purchased from Isotec-Sigma Aldrich) and carried out as a mentioned above for gross N mineralization. As in the case of gross N mineralization a reduction-microdiffusion method was used to isolate nitrate-N after conversion by Devarda’s alloy to ammonium-N from the extract into acid traps [92], [94] and nitrate concentrations were determined before starting the microdiffusions. In a first step ammonium was eliminated from the extracts by adding one acid trap and MgO (100 mg) to 10 ml sample, then the vials were closed and microdiffusion proceeded for three days at room temperature. After retrieving this acid trap, a new one was added together with 50 mg Devarda’s alloy (45% aluminum, 50% copper and 5% zinc), a reducing catalyst converting nitrate to ammonium, to the soil extracts. After five days of microdiffusion at room temperature to collect the ammonia produced from nitrate the second set of acid traps was treated as mentioned above for gross N mineralization and isotope ratios measured via EA-IRMS (see below) to estimate gross nitrification and gross nitrate immobilization.

The organic N-IPD assays described below are novel and were applied here for the first time to measure gross protein and free amino acid fluxes in soils (but see Wanek et al. [32] for the first amino acid IPD by gas chromatography-mass spectrometry).

#### Gross amino acid production and immobilization

The free amino acid pool was labeled by addition of a ^15^N-labeled algal amino acid mixture (U-^15^N-98%, Cambridge Isotope Laboratories Europe, Radeberg, Germany). For this, each 4 g fresh soil were weighed into 50 ml HDPE centrifuge tubes and the ^15^N tracer was added. Samples were extracted for half an hour with 30 ml 0.5 M K_2_SO_4_ after 5 and 30 minutes incubation time. An ultrafiltration method was used to separate low-molecular weight organic N from high molecular weight organic N. Amicon Ultra-2 pre-launch centrifugal filter devices (Millipore Corporation) with a nominal molecular weight limit (NMWL) of 3 kDa were taken to collect the low molecular weight organic N fraction (mainly amino acids and oligopeptides) together with the inorganic N forms (NO_3_^−^ and NH_4_^+^) in the permeate and the high molecular weight organic N forms (mainly proteins) in the retentate. Ultrafiltration was performed on a Beckman centrifuge (Beckman J2–21, Beckmann Coulter, Krefeld, Germany). From the labeled amino acid extract aliquots of 1.7 ml were centrifuged at 4000*g* for one hour at room temperature with a swinging bucket rotor (Beckman JS-13.1). In order to get rid of inorganic N from low molecular weight organic N, each 1 ml of permeate was filled into a 2 ml cryo-vial and 20 mg Devarda’s and 20 mg MgO were added together with an acid trap. The closed cryo-vials were kept at 37°C for 3 days; thereafter the acid traps were discarded. Via alkaline persulfate oxidation and VCl_3_/azide reaction the low molecular weight organic N was then converted to NO_3_^−^ and finally to N_2_O for analysis by PT-IRMS (see below). Moreover free amino acid concentrations were determined in each extract using the modified OPAME procedure as detailed above, to correct the isotope enrichment of free amino acids for the contributions of other low molecular weight organic N forms using a simple two-source isotope pool mixing model [95]. Free amino acid concentrations and the corrected isotope enrichments were used for later pool dilution calculations.

#### Gross protein production and consumption

Soil labeling was performed as in the case of the amino acid-IPD assays but with the ^15^N tracer being a purified algal crude protein (98 at%^15^N purchased from Isotec-Sigma Aldrich) and with incubation times of 5 min, 30 min, 4 and 24 hours. After ultrafiltration (similar to amino acid-IPD but centrifugation parameters altered) of each 1.7 ml of the labeled extract at 7500 *g* for one hour at room temperature with a fixed angle rotor (Beckman JA-20), the permeate was put aside. The high molecular weight organic N containing retentate was washed twice with each 1 ml 50 mM K_2_SO_4_ via 30 min centrifugation at room temperature and 7500 *g*, to remove inorganic N and low molecular weight organic N. Thereafter the retentate was recovered by 2 min centrifugation at 1000 *g* at room temperature and diluted to 0.5 ml with 50 mM K_2_SO_4_. The alkaline persulfate oxidation and subsequent VCl_3_/azide reactions were then used to convert the high molecular weight organic N via NO_3_^−^ to N_2_O for analysis by PT-IRMS (see below).

#### Alkaline persulfate oxidation (APO) of N_org_ (NH_4_^+^) to NO_3_^−^

The APO reaction was used to convert ammonium and organic N forms into nitrate [93]. At elevated temperature persulfate decomposes forming persulfate radicals that efficiently oxidize organic and inorganic compounds, in the case of organic N and ammonium producing nitrate. The persulfate reagent was prepared by dissolving 44 g Na_2_S_2_O_8_, 16.8 g NaOH and 30 g H_3_BO_3_ in deionized water with an end volume of 1 L [96]. For digestion aliquots of 750 µl of sample were pipetted into 1.5 ml HPLC glass vials and each 750 µl of persulfate solution were added. Vials were closed immediately (to avoid losses of ammonium due to the alkaline pH) and autoclaved for one hour at 120°C. Digested samples were stored at 4°C until further analyses.

#### VCl_3_/azide conversion of NO_3_^−^ to N_2_O

The isotope ratios of nitrate after persulfate digestion were measured by conversion of nitrate to N_2_O by a coupled VCl_3_/azide reaction in which in a first step, VCl_3_ is used to reduce nitrate to nitrite, which in a second step is further reduced to N_2_O by sodium azide [93]. For this, aliquots of 1 ml sample were pipetted into 12-ml screw cap exetainers with butyl rubber septum (IVA-VC309; IVA Analysetechnik, Merbusch, Germany) which were closed tightly and purged with helium for 10 min to get rid of air-N_2_O in the headspace. Thereafter 160 µl sodium azide and finally 1 ml VCl_3_ were added using gas-tight syringes by injection through the septa. Exetainers were put on a rotary shaker and kept at 37°C for 18 hours. The reaction was stopped by neutralizing and deactivating the sodium azide through injecting each 160 µl 6 M NaOH into the exetainers and N_2_O was analyzed by PT-IRMS.

#### PT-IRMS

The exetainer vials were then loaded into a 96-slot autosampler with a double-hole needle (GC-PAL, CTC Analytics, Zwingen, Switzerland) connected via a Gasbench II headspace analyzer (Thermo Fisher, Bremen, Germany) and a PreCon cryotrapping device (Thermo Fisher) to a gas isotope ratio mass spectrometer (IRMS, Delta V Advantage, Thermo Fisher) [93]. Through the double needle of the autosampler an auxiliary helium flow purged the contents of the exetainer vials through a water and CO_2_ scrubber into a sequence of two cryo-traps held at liquid N_2_ temperature, and the N_2_O was then released and residual CO_2_ and air (O_2_, N_2_) separated by gas chromatography. The purified sample N_2_O was then transferred into the mass spectrometer via an open split with repeated pulses of standard N_2_O gas for internal calibration (Air Liquide, Vienna, Austria). For calibration of at%^15^N respective standards varying in ^15^N:^14^N ratios were run throughout the sample preparation methods. For further details on PT-IRMS analyses see Lachouani et al. [93].

#### EA-IRMS

Acid traps and homogenized dry soils were transferred into tin capsules and directly analyzed for N content and at%^15^N by EA-IRMS. The system consisted of an elemental analyzer (EA 1110, CE Instruments, Milan, Italy) connected via a ConFlo III interface (Thermo Fisher) to the IRMS (Delta^PLUS^, Thermo Fisher). External calibration was performed using laboratory standards for isotope composition and N concentration [93].

#### Isotope pool dilution calculations

Gross production (GP) and gross consumption (GC) rates, i.e. influx into and efflux from the target pools that was labeled with ^15^N in the course of IPD experiments was then calculated based on the isotope pool dilution equations developed by others [97]:
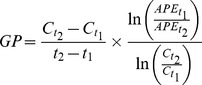


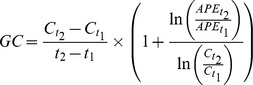
where C_t1_, C_t2_, APE_t1_ and APE_t2_ are the concentrations of N (µg N g^−1^ soil dry weight) and atom percent excess of ^15^N (APE, in %) in the respective target N pool at t_1_ and t_2_ (days), i.e. the times of IPD sample termination. APE was calculated as the difference between the at%^15^N of the given N pool at time t and the background ^15^N abundance (at%^15^N) measured in separate non-labeled samples. Gross rates are therefore expressed in µg N g^−1^ soil dry weight d^−1^.

#### Extraction of nucleic acids and quantitative PCR analyses

Nucleic acids were extracted from soil samples (0.5 g fresh weight) using a modification of the method described by Griffiths et al. 2000 [98]. Samples were loaded in 2 mL Lysing Matrix E tubes (MP Biomedicals, Illkirch, France) and cells were disrupted by bead-beating with 0.5 mL of an SDS-based lysis buffer (1% SDS, 0.7 M NaCl, 0.1 M Na_2_(SO_3_), 0.05 M EDTA pH8 and 0.1 M Tris/HCl pH 7.5) and 0.5 mL of a phenol:chloroform:isoamyl-alcohol (25∶24∶1) mixture (pH 7.5). Precipitation of nucleic acids was performed for 1 h at room temperature with 20 µg glycogen (MBI Fermentas, Vienna, Austria), 0.5 mL isopropanol and 40 µL 5 M NaCl. Precipitated nucleic acids were washed twice in 500 µL ice-cold 70% ethanol, re-suspended in 50 µL DEPC-treated water and purified by filtration through PVPP mini-columns centrifuged at 1000×*g* for 2 min at 10°C. Purity and concentration of purified nucleic acids was checked spectrophotometrically using a NanoDrop spectrophotometer (Thermo scientific) and their integrity was determined by agarose gel electrophoresis. A SybrGreen I quantitative PCR approach was used to quantify thaumarchaeal and bacterial *amoA* gene abundance. Thaumarchaeal *amoA* genes were amplified with PCR primers Arch-amoA-104F/Arch-amoA-616R [99] and bacterial *amoA* genes with primers amoA-1F/amoA-2R [100]. Duplicate dilution series (10^2^–10^7^ copies) of PCR products obtained with M13F and M13R vector primers from Topo® TA cloning® vectors (Invitrogen) containing either a 632 bp fragment of *Nitrososphaera viennensis* EN76 [101] *amoA* gene, obtained with PCR primers Arch-amoA-7F/Arch-amoA-638R [99], or a 491 bp fragment of *Nitrosospira multiformis* ATCC 25196 *amoA1* gene (Nmul_A0799/Nmul_A2325), obtained with primers amoA-1F/amoA-2R, were used as standards for the thaumarchaeal and bacterial *amoA* assays, respectively. Thaumarchaeal *amoA* standards were amplified with an efficiency of 93% and *r^2^* value of 0.997 and bacterial *amoA* standards with an efficiency of 93% and *r^2^* value of 0.991. All reactions (20 µL) contained 10 µL of 2× Quantifast™ SYBR® Green PCR Master Mix (Qiagen, Vienna, Austria), 0.2 mg.mL^−1^ BSA, 1 (Archaea) or 0.8 (Bacteria) µM of each primer and for the analysis of soil samples 10 ng of purified nucleic acid extracts. Amplification conditions were 95°C for 15 min, followed by 40 cycles of 95°C for 15 s, 60°C for 30 s (combined annealing and extension step) and plate read at 78°C for thaumarchaeal *amoA* genes and 35 cycles of 95°C for 15 s, 62°C for 30 s and plate read at 82°C for bacterial *amoA* genes, before a final extension step at 60°C for 10 min. Reactions were carried out in a Mastercycler® RealPlex^2^ thermocycler (Eppendorf Austria GmbH, Vienna, Austria) and the specificity of the amplification was assessed by melting curve analysis and agarose gel electrophoresis.

#### Statistical analyses

Mixed between-within subjects ANOVAs (split-plot ANOVA) followed by Tukey HSD post hoc tests were performed in SPSS version 16.0 for Windows (SPSS Inc., USA) to test for the effects of treatment and sampling time on biological and physicochemical soil parameters. If normality (Shapiro-Wilk test) and variance homogeneity (Levene’s test) were violated, data were log, square root or reciprocal transformed to meet these assumptions. Mauchly’s test was used to test the assumption of sphericity and if significant, the degrees of freedom of the F-distribution were corrected using the Greenhouse-Geisser procedure as the estimated epsilon was always less than 0.75. Biochar effects on pools and processes that were only measured once were tested by oneway ANOVA. Regressions and correlations were calculated with Statgraphics Centurion XVI (StatPoint Technologies Inc., USA). Simple least squares linear regressions were calculated to estimate the amount dependency of biochar effects, with added biochar amount as independent variable. Correlations between microbial community structure parameters, or with nitrification rates were performed based on the Pearson product-moment correlation procedure.

## Supporting Information

Figure S1 **Structure of beech wood biochar shown by SEM analysis.** Cross section across early to late wood transect in beech wood charcoal (top left), longitudinal section through beech charcoal showing open xylem vessels and xylem parenchyma cells (top right), close-up of xylem pits (bottom left) and close-up of a plasmodesmata channel (bottom right).
(DOCX)

Figure S2 **Scheme of the workflow for soil sample preparations to measure organic and inorganic N transformation rates using ^15^N isotope pool dilution approaches.**
(DOCX)

Table S1 **Results of ANOVA of soil physicochemical factors and gross nitrification rates for the factors treatment and sampling date for all four treatments.**
(DOCX)

Table S2 **Soil sampling dates, treatments sampled and measurements done on these samples.**
(DOCX)
