# Supplementary material for: Biochar Decelerates Soil Organic Nitrogen Cycling but Stimulates Soil Nitrification in a Temperate Arable Field Trial
Source: PLoS One. 2014 Jan 30;9(1):e86388. doi: 10.1371/journal.pone.0086388 (PMC3907405; doi:10.1371/journal.pone.0086388)
Supplement: Figure S1 — Structure of beech wood biochar shown by SEM analysis. Cross section across early to late wood transect in beech wood charcoal (top left), longitudinal section through beech charcoal showing open xylem vessels and xylem parenchyma cells (top right), close-up of xylem pits (bottom left) and close-up of a plasmodesmata channel (bottom right). (DOCX) [file pone.0086388.s001.docx]

**Figures S1. Structure of beech wood biochar shown by SEM analysis.** Cross section across early to late wood transect in beech wood charcoal (top left), longitudinal section through beech charcoal showing open xylem vessels and xylem parenchyma cells (top right), close-up of xylem pits (bottom left) and close-up of a plasmodesmata channel (bottom right).

| **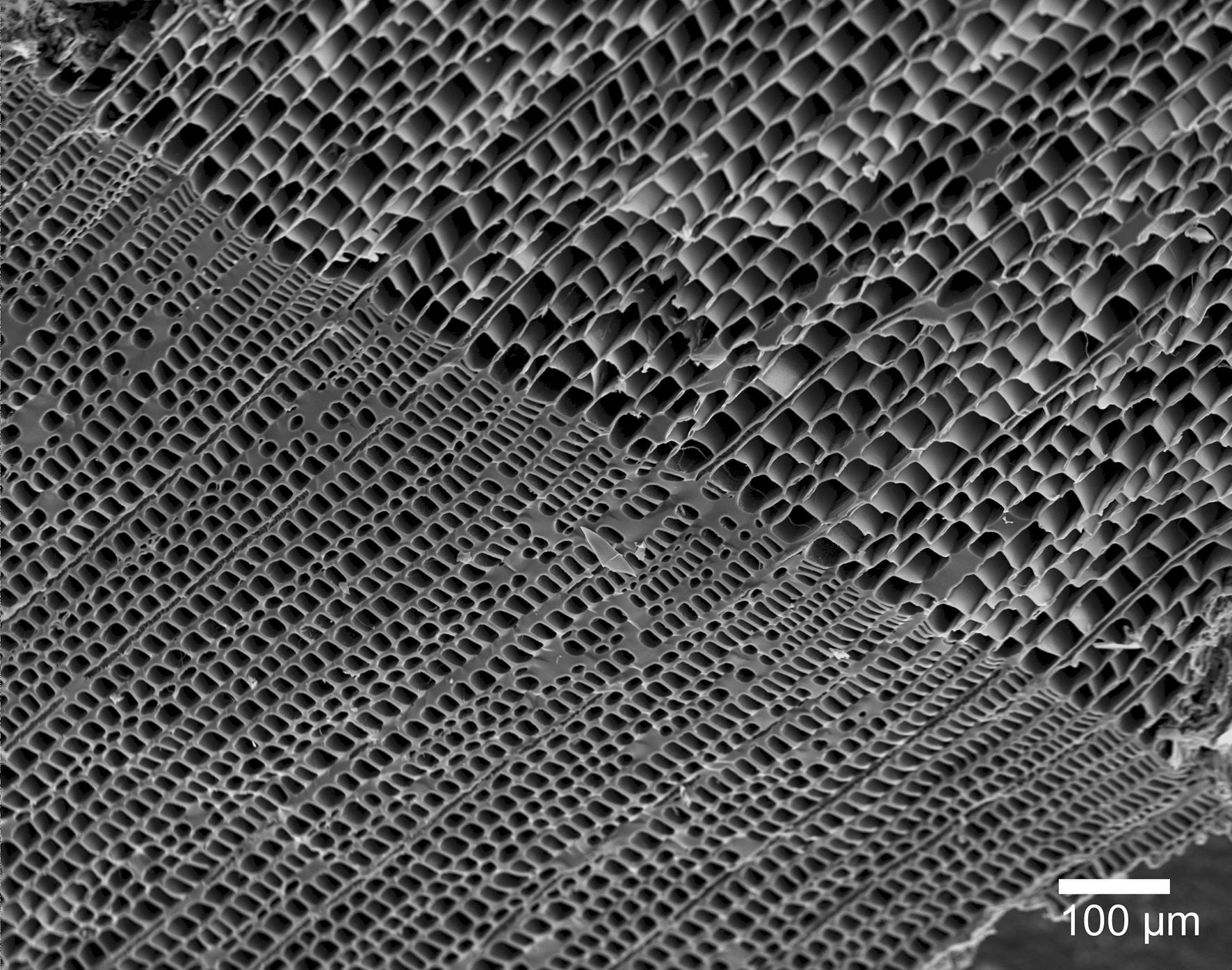** | **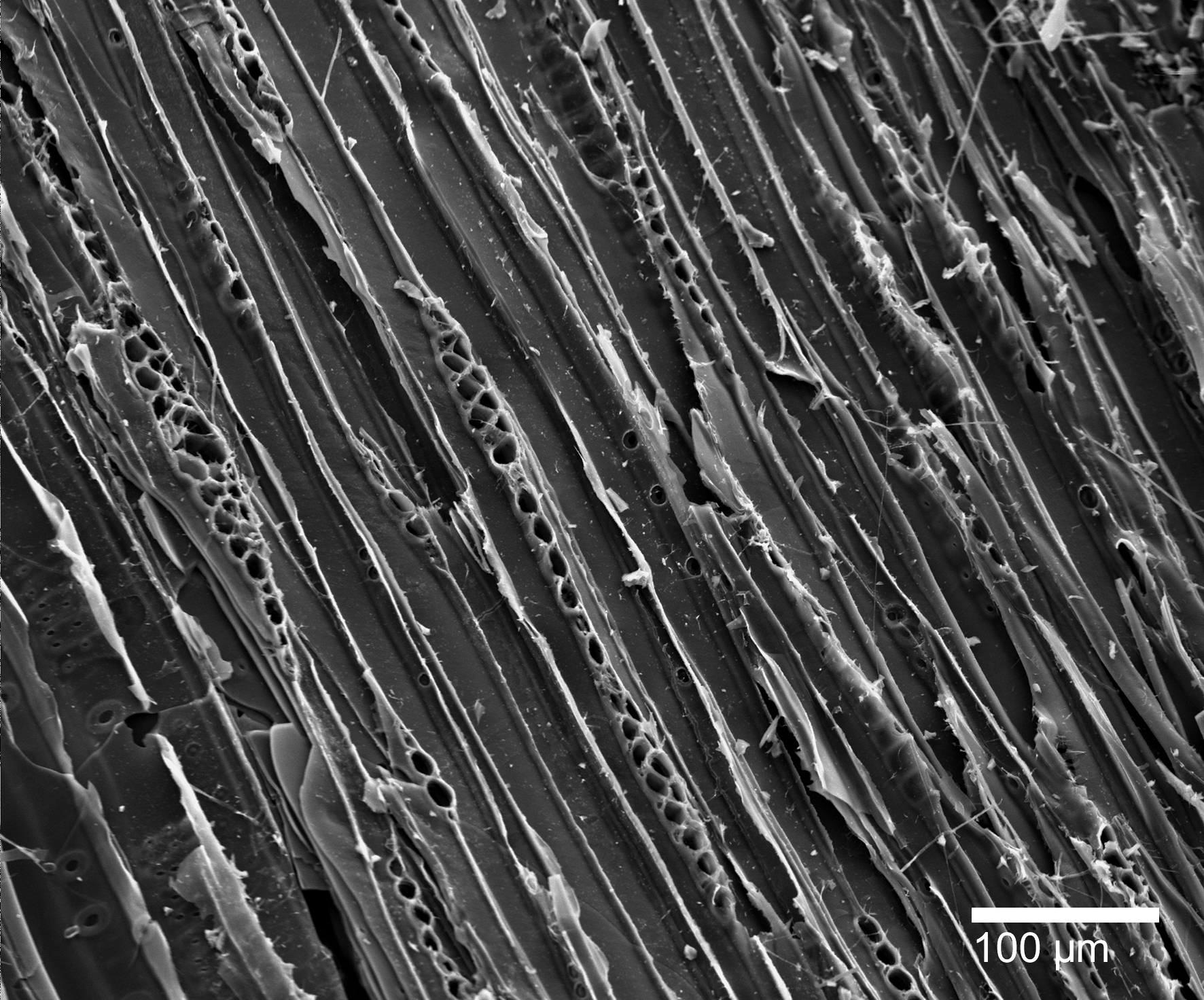** |
| --- | --- |
| **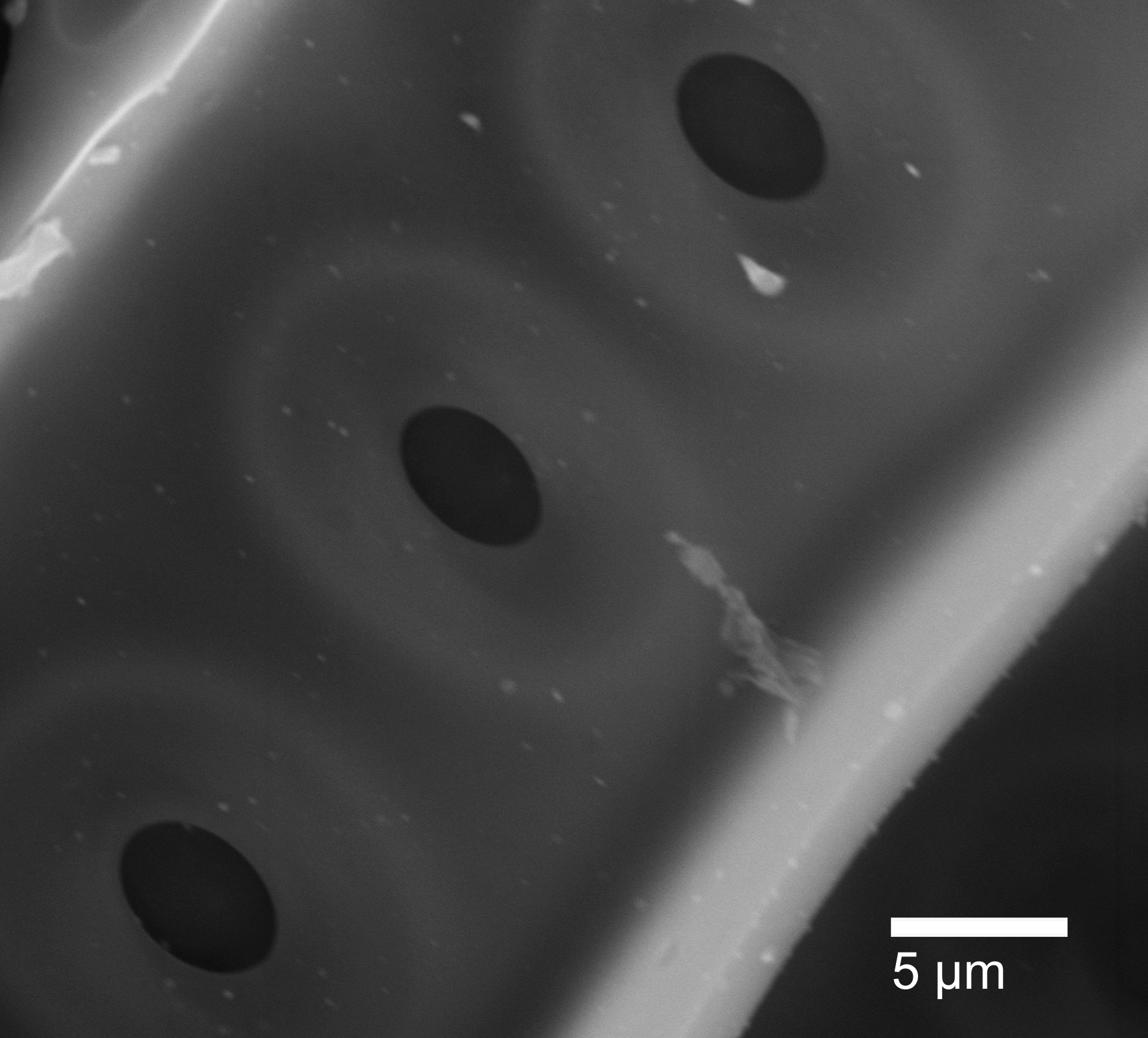** | **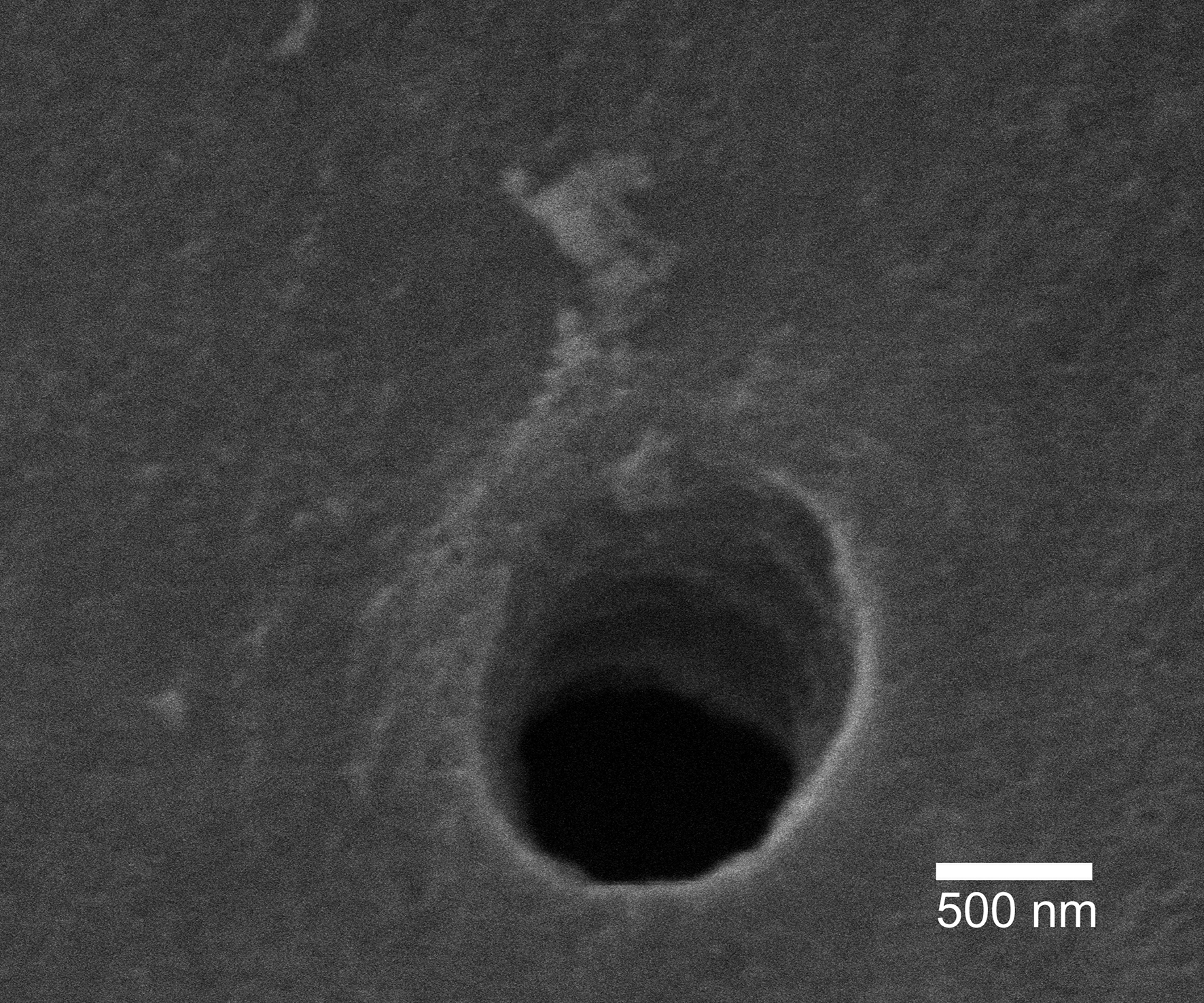** |
