# Supplementary material for: Biochar Decelerates Soil Organic Nitrogen Cycling but Stimulates Soil Nitrification in a Temperate Arable Field Trial
Source: PLoS One. 2014 Jan 30;9(1):e86388. doi: 10.1371/journal.pone.0086388 (PMC3907405; doi:10.1371/journal.pone.0086388)
Supplement: Figure S2 — Scheme of the workflow for soil sample preparations to measure organic and inorganic N transformation rates using 15N isotope pool dilution approaches. (DOCX) [file pone.0086388.s002.docx]

**Figure S2. Scheme of the workflow for soil sample preparations to measure organic and inorganic N transformation rates using ^15^N isotope pool dilution approaches.** Abbreviations: APO – alkaline persulfate oxidation, EA-IRMS – elemental analyzer isotope ratio mass spectrometry, DIN – dissolved inorganic N, DON – dissolved organic N, MD – microdiffusion, PT-IRMS – purge-and-trap isotope ratio mass spectrometry. * indicates that some N mineralization samples were to low in N for direct measurement via EA-IRMS and therefore ammonium was collected via MD, digested via APO to nitrate and then converted via VCl_3_/azide reaction to N_2_O, to be measured via PT-IRMS.
