# Supplementary material for: Biochar Decelerates Soil Organic Nitrogen Cycling but Stimulates Soil Nitrification in a Temperate Arable Field Trial
Source: PLoS One. 2014 Jan 30;9(1):e86388. doi: 10.1371/journal.pone.0086388 (PMC3907405; doi:10.1371/journal.pone.0086388)
Supplement: Table S1 — Results of ANOVA of soil physicochemical factors and gross nitrification rates for the factors treatment and sampling date for all four treatments. (DOCX) [file pone.0086388.s003.docx]

**Table S1. Results of ANOVA of soil physicochemical factors and gross nitrification rates for the factors treatment and sampling date for all four treatments.**

|  | NPK |  | BC1N |  | BC3 |  | BC3N |  | treatment | | time | | interaction | |
| --- | --- | --- | --- | --- | --- | --- | --- | --- | --- | --- | --- | --- | --- | --- |
| pools |  |  |  |  |  |  |  |  | F | P | F | P | F | P |
| C_org_ | 18.66 | a | 28.91 | b | 57.36 | c | 47.43 | c | (3, 12) 96.2 | < 0.001 | (1, 12) 1.7 | 0.216 | (3, 12) 4.2 | 0.031 |
| N_tot_ | 1.40 |  | 1.48 |  | 1.54 |  | 1.58 |  | (3, 12) 1.8 | 0.195 | (1, 12) 155.5 | < 0.001 | (3, 12) 1.0 | 0.424 |
| BD | 1.17 | b | 1.16 | b | 1.06 | ab | 1.04 | a | (3, 12) 5.7 | 0.012 | (1, 12) 96.9 | < 0.001 | (3, 12) 1.0 | 0.447 |
| Grav. WC | 0.13 | a | 0.14 | a | 0.15 | b | 0.15 | b | (3, 12) 13.2 | < 0.001 | (5, 60) 250.9 | < 0.001 | (15, 60) 3.4 | < 0.001 |
| Porosity | 56.03 | a | 56.13 | a | 60.06 | ab | 60.61 | b | (3, 12) 5.7 | 0.012 | (1, 12) 96.8 | < 0.001 | (3, 12) 0.9 | 0.449 |
| WFPS | 26.75 | ab | 27.96 | b | 25.89 | ab | 25.82 | a | (3, 12) 3.8 | 0.039 | (5, 60) 248.8 | < 0.001 | (15, 60) 1.7 | 0.074 |
| AFPS | 73.26 | ab | 72.04 | a | 74.11 | ab | 74.18 | b | (3, 12) 3.8 | 0.039 | (5, 60) 248.8 | < 0.001 | (15, 60) 1.7 | 0.074 |
| DOC | 122.56 | b | 118.71 | b | 99.32 | a | 111.21 | ab | (3, 12) 9.4 | 0.002 | (2.7, 32.5) 297.8 | < 0.001 | (8.1, 32.5) 1.7 | 0.128 |
| DON | 9.09 |  | 8.43 |  | 7.29 |  | 8.22 |  | (3, 12) 1.9 | 0.180 | (1.2, 14.5) 70.1 | < 0.001 | (3.6, 14.5) 1.7 | 0.210 |
| DOC:DON | 16.25 | a | 30.00 | b | 18.55 | a | 15.92 | a | (3, 12) 8.8 | 0.002 | (1.3, 15.3) 23.6 | < 0.001 | (3.8, 15.3) 5.5 | 0.007 |
| NO_3_^-^ | 13.66 | c | 13.25 | c | 4.33 | a | 7.21 | b | (3, 12) 37.8 | < 0.001 | (5, 60) 267.6 | < 0.001 | (15, 60) 9.2 | < 0.001 |
| NH_4_^+^ | 1.74 | ab | 2.17 | b | 1.55 | ab | 1.61 | a | (3, 12) 4.6 | 0.023 | (1.9, 22.5) 62.8 | < 0.001 | (5.6, 22.5) 2.3 | 0.072 |
| FAA | 0.77 | b | 0.73 | ab | 0.69 | a | 0.68 | a | (3, 12) 7.3 | 0.005 | (5, 60) 17.2 | < 0.001 | (15, 60) 2.6 | 0.005 |
| TDN | 24.95 | c | 24.22 | c | 13.17 | a | 17.03 | b | (3, 12) 21.0 | < 0.001 | (1.7, 19.8) 192.0 | < 0.001 | (5.0, 19.8) 2.0 | 0.122 |
| DNA | 12.33 |  | 16.86 |  | 13.18 |  | 17.47 |  | (3, 12) 2.4 | 0.118 | (1, 12) 2.0 | 0.184 | (3, 12) 2.3 | 0.132 |
| AOA/DNA | 1.16E6 |  | 1.15E6 |  | 1.18E6 |  | 1.31E6 |  | (3, 12) 1.4 | 0.285 | (1, 12) 24.0 | < 0.001 | (3, 12) 1.5 | 0.274 |
| AOA/soil | 1.35E7 | a | 1.96E7 | ab | 1.70E7 | ab | 2.34E7 | b | (3, 12) 3.8 | 0.039 | (1, 12) 9.6 | 0.009 | (3, 12) 0.6 | 0.600 |
| AOB/DNA | 3.28E5 |  | 4.76E5 |  | 3.68E5 |  | 4.44E5 |  | (3, 12) 3.2 | 0.061 | (1, 12) 30.6 | < 0.001 | (3, 12) 2.5 | 0.106 |
| AOB/soil | 3.91E6 | a | 8.50E6 | b | 5.24E6 | ab | 7.89E6 | b | (3, 12) 3.9 | 0.037 | (1, 12) 8.719 | 0.012 | (3, 12) 0.6 | 0.656 |
| AOA:AOB | 3.83 |  | 2.64 |  | 3.23 |  | 3.34 |  | (3, 12) 1.7 | 0.223 | (1, 12) 0.284 | 0.604 | (3, 12) 0.1 | 0.962 |
| Gross_nit | 3.48 | a | 6.49 | b | 7.58 | bc | 8.85 | c | 21.54 | <0.001 |  |  |  |  |
| NO_3_^-^_GC | 4.62 |  | 7.39 |  | 9.22 |  | 8.95 |  | 3.2 | 0.062 |  |  |  |  |

Data were analyzed by two-way mixed ANOVA, and were transformed (e.g. log, sqrt, reciprocal) if necessary to meet the criteria of ANOVA, normality and variance homogeneity. Data for gross nit and NO_3_^-^_GC were available only for September 2012 and therefore analyzed by one-way ANOVA. Abbreviations: C_org_, soil organic C; N_tot_, total soil N; BD, soil bulk density; WC, gravimetric soil water content; porosity, percent pore space; WFPS, percent water filled pore space; AFPS, percent air filled pore space; DOC, dissolved organic C; DON, dissolved organic N; NO_3_^-^, nitrate; NH_4_^+^, ammonium; FAA, total free amino acids; TDN, total dissolved N; Gross_nit, gross nitrification; NO_3_^-^_GC, gross nitrate consumption; DNA, soil DNA content; AOA/DNA, archaeal amoA copy numbers on DNA basis; AOA/soil, archaeal amoA copy numbers on dry soil basis; AOB/DNA, bacterial amoA copy numbers on DNA basis; AOB/soil, bacterial amoA copy numbers on dry soil basis;.
