# Supplementary material for: Biochar Decelerates Soil Organic Nitrogen Cycling but Stimulates Soil Nitrification in a Temperate Arable Field Trial
Source: PLoS One. 2014 Jan 30;9(1):e86388. doi: 10.1371/journal.pone.0086388 (PMC3907405; doi:10.1371/journal.pone.0086388)
Supplement: Table S2 — Soil sampling dates, treatments sampled and measurements done on these samples. (DOCX) [file pone.0086388.s004.docx]

**Table S2. Soil sampling dates, treatments sampled and measurements done on these samples.**

| **Parameter** | **Jul 2011** | **Sep 2011** | **Dec 2011** | **Jan 2012** | **May 2012** | **Jun 2012** | **Jul 2012** | **Aug 2012** | **Sep 2012** |
| --- | --- | --- | --- | --- | --- | --- | --- | --- | --- |
| SWC | XX | all | all |  | all |  | all | all | all |
| WFPS | XX | all | all |  | all |  | all | all | all |
| BD |  | all |  |  |  |  |  |  | all |
| C_org_ |  | all |  |  |  |  |  |  | all |
| N_tot_ | XX | all |  |  |  |  |  |  | all |
| DON/DOC | XX | all | all |  | all |  | all | all | all |
| DIN/FAA | XX | all | all |  | all |  | all | all | all |
| Protein/UF | XX |  |  |  |  |  |  |  |  |
| IPD-array | XX |  |  |  |  |  |  |  |  |
| IPD-Nitrate | XX |  |  |  |  | XX |  |  | all |
| AOA/AOB |  |  |  | all |  | XX |  |  | all |
| Soil other | XX |  |  |  |  |  |  |  |  |
| Biochar | XX |  |  |  |  |  |  |  |  |

The field experiment was setup in March 2011. Treatments sampled: XX, control (NPK) and biochar (BC3N) plots; all, all four treatments i.e. NPK, BC1N, BC3 and BC3N. Parameters measured: SWC, soil water content; WFPS, water filled pore space; BD, soil bulk density; Corg, soil organic C; Ntot, total soil N; DON/DOC, dissolved organic N and dissolved organic C; DIN/FAA, DIN (ammonium and nitrate) and free amino acids; Protein/UF, protein and high- and low molecular weight organic N by ultrafiltration; IPD-array, all organic N and inorganic N transformation rates by isotope pool dilution; IPD-nitrate, nitrate transformation rates by isotope pool dilution; AOA/AOB, soil DNA content, archaeal and bacterial amoA abundances; Soil other, soil characterisation for soil texture, cation-exchange capacity, base saturation etc.; Biochar, biochar characterisation for pore space and element contents.
